# Ubiquitin-like protein 3 (UBL3) is required for MARCH ubiquitination of major histocompatibility complex class II and CD86

**DOI:** 10.1038/s41467-022-29524-w

**Published:** 2022-04-11

**Authors:** Haiyin Liu, Kayla R. Wilson, Ashley M. Firth, Christophe Macri, Patrick Schriek, Annabelle B. Blum, Javiera Villar, Samuel Wormald, Mitch Shambrook, Bangyan Xu, Hui Jing Lim, Hamish E. G. McWilliam, Andrew F. Hill, Laura E. Edgington-Mitchell, Irina Caminschi, Mireille H. Lahoud, Elodie Segura, Marco J. Herold, Jose A. Villadangos, Justine D. Mintern

**Affiliations:** 1 Department of Biochemistry and Pharmacology, The University of Melbourne, Bio21 Molecular Science and Biotechnology Institute, 30 Flemington Rd, Parkville, VIC 3010 Australia; 2 Institut Curie, PSL Research University, INSERM, U932, 26 rue d’Ulm, 75005 Paris, France; 3 The Walter and Eliza Hall Institute of Medical Research, Parkville, VIC 3010 Australia; 4 Department of Biochemistry and Genetics, La Trobe Institute for Molecular Science, La Trobe University, Bundoora, VIC 3086 Australia; 5 Department of Microbiology and Immunology, Peter Doherty Institute for Infection and Immunity, The University of Melbourne, Parkville, VIC 3010 Australia; 6 Department of Oral and Maxillofacial Surgery, Bluestone Center for Clinical Research, New York University College of Dentistry, New York, NY 10010 USA; 7 Drug Discovery Biology, Monash Institute of Pharmaceutical Sciences, Monash University, Parkville, VIC 3052 Australia; 8 Monash Biomedicine Discovery Institute and Department of Biochemistry and Molecular Biology, Monash University, Clayton, VIC 3800 Australia; 9 Department of Medical Biology, University of Melbourne, Parkville, VIC 3010 Australia

**Keywords:** Ubiquitylation, Antigen-presenting cells, MHC class II, Conventional dendritic cells

## Abstract

The MARCH E3 ubiquitin (Ub) ligase MARCH1 regulates trafficking of major histocompatibility complex class II (MHC II) and CD86, molecules of critical importance to immunity. Here we show, using a genome-wide CRISPR knockout screen, that ubiquitin-like protein 3 (UBL3) is a necessary component of ubiquitination-mediated trafficking of these molecules in mice and in humans. *Ubl3*-deficient mice have elevated MHC II and CD86 expression on the surface of professional and atypical antigen presenting cells. UBL3 also regulates MHC II and CD86 in human dendritic cells (DCs) and macrophages. UBL3 impacts ubiquitination of MARCH1 substrates, a mechanism that requires UBL3 plasma membrane anchoring via prenylation. Loss of UBL3 alters adaptive immunity with impaired development of thymic regulatory T cells, loss of conventional type 1 DCs, increased number of trogocytic marginal zone B cells, and defective in vivo MHC II and MHC I antigen presentation. In summary, we identify UBL3 as a conserved, critical factor in MARCH1-mediated ubiquitination with important roles in immune responses.

## Abstract (web summary)

Regulated trafficking of major histocompatibility complex class II and CD86 is a prerequisite of antigen presenting cell functionality. Authors show here that ubiquitin-like protein 3 is critically involved in the ubiquitination process that controls trafficking, with wide-ranging immunological consequences.

## Introduction

Ubiquitination controls the function of target proteins through a series of reactions involving E1-activating, E2-conjugating, and E3-ligating enzymes^1^. The E3 family of membrane-associated RING-CH (MARCH) E3 ligases regulates key immunoreceptors including major histocompatibility complex class II (MHC II)^2–6^ and CD86^7^. Ubiquitination of peptide-loaded MHC II complexes affects their trafficking, prevents their recycling from endosomes to the plasma membrane, and promotes their endosomal degradation^8^, therefore reducing their cell surface abundance. This process is of critical importance in several aspects of adaptive immunity. For example, MHC II regulation in thymic epithelial cells (TEC) by MARCH8 affects CD4^+^ T-cell thymic development^5,6^ while lack of MHC II ubiquitination in thymic conventional DCs (cDCs) by MARCH1 disrupts regulatory T (T_reg_) cell selection^9^. MARCH1 deficiency also causes high CD86 and MHC II expression in other conventional and non-conventional antigen-presenting cells^10^, alters surface expression of multiple immune molecules including MHC I^11,12^, induces marginal zone B-cell trogocytosis of cDC membrane^13^, and causes reductions in splenic cDC numbers^13–15^. Many other immunoreceptors are reportedly regulated by the MARCH family, though only MHC II and CD86 have been unequivocally identified as targets of MARCH ubiquitination in vivo^10,16^.

Little is known about the machinery involved in MARCH ubiquitination. For example, it is not known whether MARCH1 or MARCH8 cooperate with specific E2 enzymes, require adaptor proteins, or whether their ubiquitination is modulated by deubiquitinases. One E2, UBE2D1, has been shown to regulate MARCH1 expression, but there is no evidence it participates in MARCH1-mediated substrate ubiquitination^17^. So far, the only protein reported to affect MHC II ubiquitination is CD83^18,19^, a membrane protein that associates with the transmembrane domains of MARCH1 and MARCH8 thereby blocking its interaction with substrates^19^.

Here, using a whole-genome CRISPR/Cas9 knockout screen, we identify ubiquitin-like protein 3 (UBL3) as a novel regulator of MHC II, via its role in MARCH1-mediated ubiquitination. We report that UBL3 regulates MARCH1 substrates MHC II and CD86 in an array of mouse and human immune cells, is important for maintaining cDC homeostasis, impacts cDC antigen presentation, and is required for T_reg_ development. These results reveal the first immunological role for UBL3.

## Results

### Identification of candidate genes regulating MARCH ubiquitination using a genome-wide CRISPR/Cas9 screen

**Fig. 1:**
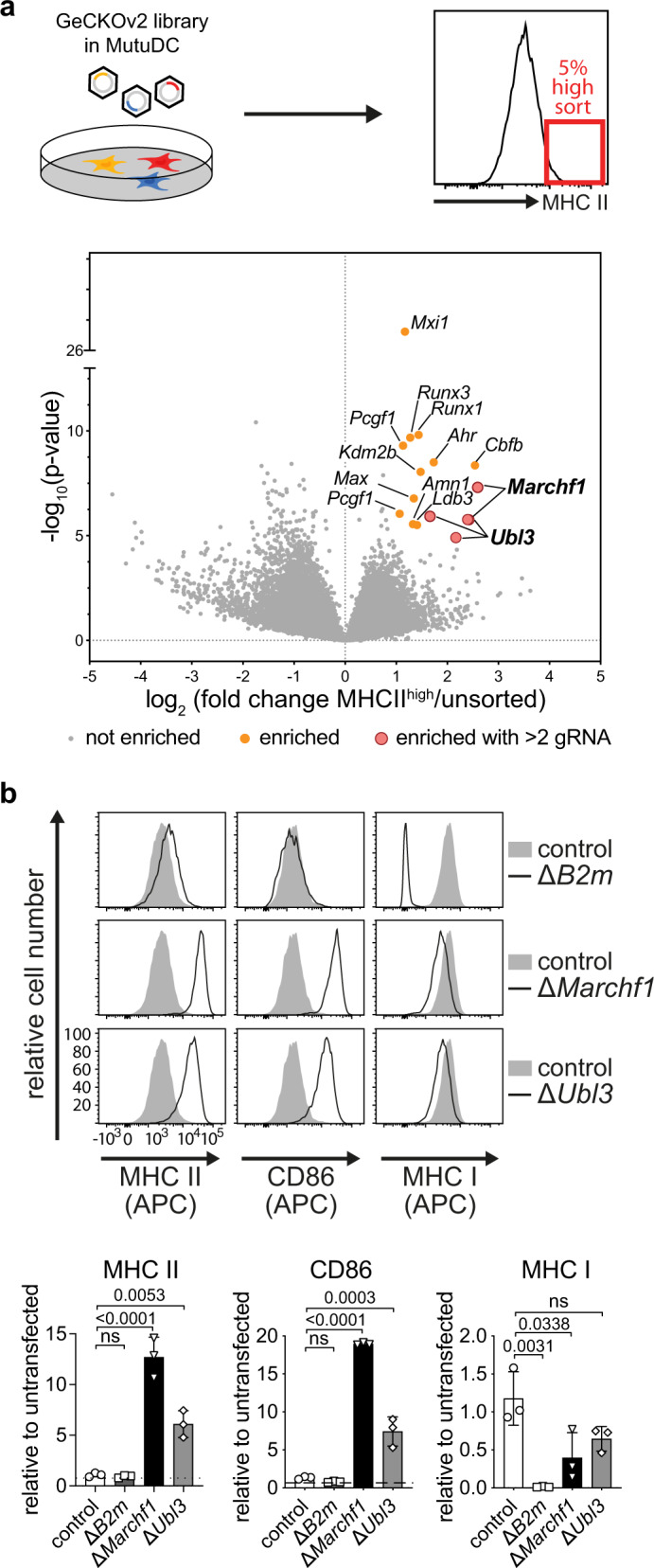
*Ubl3* regulates surface expression of MHC II and CD86 in MutuDCs. **a** Schematic of CRISPR/Cas9 genetic screen. MutuDCs were lentivirally transduced with the GeCKOv2 library A and puromycin-resistant cells were stained for surface MHC II. The 5% highest MHC II expressing cells were sorted by flow cytometry, genomic DNA extracted, and amplified. gRNAs were quantified by Illumina NextSeq sequencing and analyzed with edgeR. Volcano plots show detected gRNAs enriched in sorted cells with increased MHC II expression compared to the unsorted library. *P* values were assessed using a negative binomial generalized linear model with a two-sided likelihood ratio test, with adjustment for multiple testing using the Benjamini–Hochberg false discovery rate (FDR) method. Guide RNAs with an absolute log2 fold change >1 and FDR of <0.01 were considered significantly enriched (orange dots), with genes hit by at least two gRNAs highlighted (large red circles). **b** Surface expression of MHC II, CD86, or MHC I were analyzed by flow cytometry for MutuDCs lacking *B2m* (pool), *Marchf1,* or *Ubl3* (single-cell clones), compared with control cells expressing non-targeting *hBim* gRNA. Top: representative histograms, with gray solid histograms: control MutuDCs expressing non-targeting *hBim* gRNA, black lined histograms: CRISPR/Cas9 modified MutuDC lines as indicated. Bottom: quantification of flow cytometry analysis from three experiments. Bars indicate mean+SD of geometric mean fluorescence intensity (gMFI), relative to untransfected cells. Statistical analysis was performed using a one-way ANOVA and Bonferroni’s multiple comparisons test, comparing each sample to *hBim* controls, *p* values shown above bars, ns (not significant) *p* > 0.05.

To identify new genes in MARCH ubiquitination an unbiased, genome-wide CRISPR/Cas9 forward genetic screen was performed. Surface MHC II was used as a surrogate read out, given its surface expression increases when MARCH ubiquitination is disrupted^2–4^. We used MutuDCs^20^, which express and regulate MHC II ubiquitination using MARCH1 similar to primary DCs^11^. MutuDCs were transduced with the genome-wide CRISPR knockout (GeCKO) v2 library and surface MHC II detected by flow cytometry. Library transduced MutuDCs were stained with fluorescent anti-MHC II antibody (clone M5/114) and cells expressing the 5% highest MHC II (MHC II^high^) were isolated by flow cytometry. The gRNAs enriched in MHC II^high^ cells were considered “hits” when their count increased at least two-fold compared to the reference library (log_2_ fold change >1), with a false discovery ratio of 1% (FDR < 0.01). Twelve candidate genes were identified, with the top enriched gRNA targeting *Marchf1*, indicating the validity of the approach (Fig. 1a, Table 1). Among these candidates, UBL3 has been previously described to be associated with the ubiquitin system^21,22^ and was therefore further investigated as a potential participant in MARCH ubiquitination.

**Table 1:** Enriched gRNAs in the MHC II^high^ population.

FC	log_10_(*p* value)	Gene	Name	gRNAs
6.04	7.313	March1	E3 ubiquitin-protein ligase MARCH1	2
5.82	8.362	Cbfb	Core-binding factor subunit beta	1
3.33	8.503	Ahr	Aryl hydrocarbon receptor	1
3.16	5.923	Ubl3	Ubiquitin-like protein 3	3
2.78	8.049	Kdm2b	Lysine-specific demethylase 2B	1
2.71	9.817	Runx3	Runt-related transcription factor 3	1
2.65	5.512	Ldb3	LIM domain-binding protein 3	1
2.54	6.780	Max	Protein max	1
2.51	5.556	Amn1	Antagonist of mitotic exit network 1	1
2.42	9.681	Runx1	Runt-related transcription factor 1	1
2.25	26.770	Mxi1	Max-interacting protein 1	1
2.19	9.306	Pcgf1	Polycomb group RING finger protein 1	2

*P* values were assessed using a negative binomial generalized linear model with a two-sided likelihood ratio test, with adjustment for multiple testing using the Benjamini–Hochberg false discovery rate (FDR) method. Shown are hits with false discovery rate <0.01 and >2-fold enrichment over the reference library. *FC* fold change, *gRNAs* number of gRNAs detected.

To validate UBL3 as a hit, *Ubl3*-deficient MutuDCs (Δ*Ubl3*) were generated using an inducible CRISPR/Cas9 system, and single-cell clones were isolated. Deletion of *Marchf1* served as a positive control, while deletion of *Β2m*, the gene encoding the light chain of MHC I molecules, was used to rule out non-specific increases in surface molecules. Negative control MutuDCs were transduced with a gRNA targeting human *Bim* (*hBim*) that does not target any mouse genes^11^. Successful *Ubl3* gene deletion was confirmed by inference of CRISPR edits (ICE) sequencing of clonal cell lines^23^ (Supplementary Fig. 1). MutuDCs were examined for surface expression of MHC II and also CD86, the second known MARCH1 substrate^7,10^. Surface MHC I expression was also assessed. Δ*Ubl3* MutuDCs specifically increased surface MHC II and CD86 compared to control MutuDCs (Fig. 1b). As expected, MHC I was reduced in *Β2m*-deficient MutuDCs (Δ*B2m*), without alterations in surface MHC II or CD86 (Fig. 1b). MHC I was slightly reduced for Δ*Ubl3* MutuDCs, a phenomenon also observed for MARCH1-deficient (Δ*Marchf1)* cells^11,12^ (Fig. 1b). Taken together, these results confirm that cells lacking UBL3 expression phenocopy MARCH1 deficiency, so we examined whether UBL3 is involved in MARCH1-mediated ubiquitination.

### UBL3 does not control MHC II synthesis, assembly, or peptide loading

**Fig. 2:**
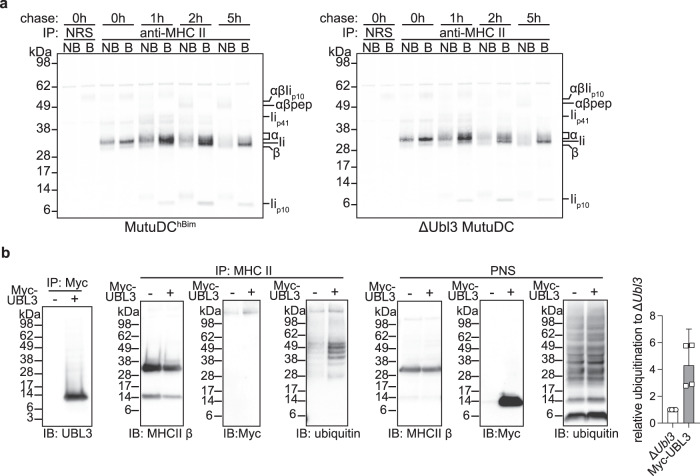
UBL3 does not control MHC II via altering synthesis, peptide loading, or UBL3 modification of MHC II. **a** Analysis of Ii degradation and MHC II peptide loading in control MutuDCs expressing non-targeting *hBim* gRNA or Δ*Ubl3* MutuDCs. Cells were metabolically labeled for 30 min, washed, and cultured in a growth medium for the indicated time points before lysis. MHC II molecules were immunoprecipitated with JV1 rabbit antisera, or normal rabbit serum (NRS), and protein G-Sepharose. Samples were loaded before (NB) or after boiling (B) at 95 °C (**b**), subjected to denaturing SDS-PAGE, transferred onto PVDF membranes, and exposed to a storage phosphor screen. Sizes corresponding to free MHC II α and β, full-length invariant chain (Ii), the Ii spliced variant Ii_p41_, the degradation intermediate Ii_p10_, and SDS-stable complexes αβIi_p10_, and αβ-peptide (pep), are indicated on the right. Data from one experiment. **b** Δ*Ubl3* MutuDCs or Δ*Ubl3* MutuDCs expressing Myc-UBL3 were lysed and post-nuclear supernatants (PNS) were subjected to immunoprecipiation (IP) with anti-Myc-Tag agarose (clone 9E10) or anti-MHC II antibody (M5/114) crosslinked to protein G-sepharose. Per lane, samples equivalent to 5 × 10^6^ cells (Myc IP), 2.5 × 10^6^ cells (MHC II IP), or 400,000 cells (PNS) were analyzed by non-denaturing SDS-PAGE and western blotting using antibodies against UBL3 (ab113820), MHC II β chain (JV2), Myc (71D10), and ubiquitin (P4D1). Shown are representative blots and quantification of relative ubiquitination, with bars displaying mean + 95% confidence intervals for four experiments.

First, we compared wild-type (WT) and UBL3-deficient MutuDC MHC II synthesis, assembly, and peptide loading, processes that have been extensively characterized and can be visualized biochemically using radioactive pulse-chase and immunoprecipitation^24–26^. Synthesis and assembly of MHC II α and β subunits with the chaperone Ii (CD74), assessed by radiolabeling and immunoprecipitation of the complex, was unaffected by the lack of UBL3 (Fig. 2a). To assess intracellular conversion of these complexes into MHC II dimers bound to antigenic peptides, “pulsed” cells were cultured in radiolabel-free medium (“chased”) and MHC II was immunoprecipitated. The immunoprecipitates were incubated at high (boiled) or room (non-boiled) temperature to visualize “SDS-stable complexes”, and loaded in reducing sodium dodecyl sulfate–polyacrylamide gel electrophoresis (SDS-PAGE) to analyze the composition of the immunoprecipitated molecules, as described in detail elsewhere^25,26^. In brief, this experiment showed the lack of UBL3 did not affect: (i) MHC II-Ii trafficking through the Golgi complex; (ii) proteolytic degradation of Ii in endosomal compartments; (iii) formation of MHC II complexes bound to the Iip10 intermediate fragment; (iv) cleavage of Iip10 by cathepsin S into the CLIP peptide; and (v) H-2DM-chaperoned substitution of CLIP with antigenic peptides. This implied that UBL3 regulated MHC II surface expression after the formation of peptide-loaded complexes and, most likely, deposition on the plasma membrane, as shown for MARCH1^8^.

### UBL3 does not bind covalently to MHC II but regulates its ubiquitination

Given that covalent binding of UBL3 has been reported as a post-translational modification that regulates target proteins^27^, we examined the possibility that MHC II was modified with UBL3. Myc-tagged UBL3 (Supplementary Fig. 1b) was retrovirally expressed in Δ*Ubl3* MutuDCs, immunoprecipitated with anti-Myc from transduced or untransduced cells, and analyzed by western blot using anti-UBL3 or anti-Myc antibodies. Consistent with published data^27^, a smear indicative of the presence of a heterogenous mix of UBL3-modified proteins of different molecular weights was visible in lanes containing immunoprecipitated Myc-UBL3 (Fig. 2b). However, immunoprecipitation of MHC II and analysis by western blot using anti-Myc showed no evidence of UBL3 modification (Fig. 2b). This implied that Myc-UBL3 was able to post-translationally modify many proteins but not MHC II. We tested the alternative hypothesis that UBL3 participated in MARCH1-mediated ubiquitination. Western blot analysis of MHC II immunoprecipitated from cells with intact MARCH1 ubiquitination machinery using anti-Ub mAb reveals a ladder of several discrete bands corresponding to isoforms of I-A_β_ covalently bound to one or more Ub^4,11^. Very little ubiquitinated MHC II was observed in UBL3-deficient MutuDCs, but expression of Myc-UBL3 significantly increased MHC II ubiquitination (Fig. 2b). We conclude that UBL3 is a regulator of MARCH1-mediated ubiquitination.

### UBL3 regulation of MHC II and CD86 surface expression requires membrane anchoring

**Fig. 3:**
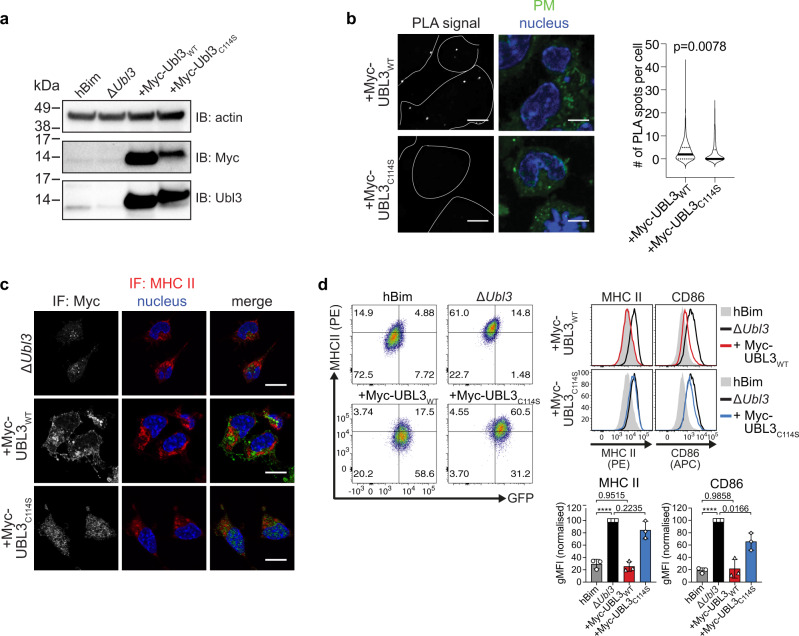
UBL3 regulation of MHC II and CD86 requires membrane anchoring. **a**–**d** Clonal Δ*Ubl3* MutuDCs were retrovirally transduced with Myc-tagged wild type UBL3 (“+Myc-UBL3_WT_”), or UBL3_C114S_ mutant (“+Myc-UBL3_C114S_”). **a** Post-nuclear supernatants of 2.5 × 10^5^ cells were analyzed by non-denaturing SDS-PAGE and western blotting using anti-actin (20-33) anti-Myc (71D10) and anti-Ubl3 (ab113820) antibodies. Representative blot is shown for two independent experiments with similar results. **b** Proximity ligation assay (PLA). Δ*Ubl3* MutuDC expressing Myc-UBL3_WT_ or Myc-UBL3_C114S_ were stained with plasma membrane (PM) CytoPainter. After washing, fixing, and permeabilization, cells were stained with Hoechst 33342, rabbit anti-MHC II (JV2) antiserum, and mouse anti-Myc mAb (9E10) and subjected to PLA. Scale bar = 10 µm. PLA spots indicate spatial proximity between MHC II and UBL3 and were enumerated within plasma membrane boundaries (violin plots). One experiment, *P* value as indicated, unpaired Welch’s *t* test (two-sided). **c** Immunofluorescence microscopy. Cells were fixed, permeabilized, and stained with biotinylated rat anti-MHC II mAb (M5/114), mouse anti-Myc mAb (9E10), streptavidin Alexa Fluor 647, donkey anti-mouse Alexa Fluor 594, and 0.5 μg/ml DAPI. Scale bar = 10 µm. Representative image shown for two independent experiments with similar results. **d** Flow cytometry analysis of MHC II and CD86. Left: Dot plots show MHC II (*x* axis) and GFP (*y* axis). Center: histograms show corresponding expression levels of MHC II or CD86, with gray solid histogram: control MutuDCs expressing non-targeting *hBim* gRNA; black line: Δ*Ubl3* MutuDCs, red line: Δ*Ubl3* MutuDCs expressing Myc-UBL3_WT_, blue line: Δ*Ubl3* MutuDCs expressing Myc-UBL3_C114S_. Right: quantification of gMFI from three experiments, normalized to highest gMFI of each experiment, with bars mean ± SD and symbols. ****P < 0.0001, one-way ANOVA with Bonferroni’s test.

MARCH1 and MARCH8 recognize their membrane receptor substrates via transmembrane region interactions^19,28,29^ and mediate ubiquitination of residues in close proximity to the inner leaflet of the membrane^16^. We hypothesized that the involvement of UBL3 in this process may also require recruitment to sites of MARCH ubiquitination. It has been previously reported that prenylation at cysteine 114 is required for UBL3 localization to the plasma membrane in plants^30^ and mammalian cells^27^, so we tested the role of this post-translational modification in MARCH function. We retrovirally transduced Δ*Ubl3* MutuDCs with myc-tagged *Ubl3* or its mutant form *Ubl3*_*C114S*_, which cannot be prenylated^31^ (Supplementary Fig. 1b). Expression of the two transduced proteins, and reduced electrophoretic mobility of prenylation-deficient Myc-UBL3_C114S_ was observed as expected (Fig. 3a)^32^. We used these cell lines to address the hypothesis that prenylated UBL3 was recruited to membrane regions where MARCH1 ubiquitinates its substrates.

Proximity ligation assays (PLA) demonstrated close association between MHC II and Myc-UBL3_WT_, visible as puncta that decreased in number, in cells expressing prenylation-deficient Myc-UBL3_C114S_ (Fig. 3b). As this suggested UBL3 was recruited to sites of MHC II ubiquitination, we next attempted to demonstrate a physical association between UBL3 and MHC II and/or MARCH1 using pull-down experiments. Lysates from MutuDCs, or MutuDCs expressing Myc-UBL3_WT_ or Myc-UBL3_C114S_ were incubated with anti-Myc agarose beads, washed, and subjected to on-bead digest. Liquid chromatography-tandem mass spectrometry recovered 1329 proteins at a false discovery rate (FDR) of 1%. We identified 17 proteins specifically immunoprecipitated from myc-UBL3-expressing cells, including UBL3 itself, with five of these also detected in the Myc-UBL3_C114S_ samples. (Supplementary Fig. 2 and Supplementary Tables 1, 2). We did not further study the role of these “hits” as none have a known role in the regulation of membrane receptor expression, though they may play a role in other UBL3 functions. Neither MHC II nor MARCH1 was pulled down with Myc-UBL3. This was not surprising because, first, MARCH1 is expressed at such low levels that it can only be detected in transfected cells, which are unsuitable to study interactions with UBL3 because when MARCH1 is overexpressed it interacts with, and ubiquitinates, non-physiological substrates^10,16^. Second, any interaction between UBL3 and MARCH1 or MHC II is probably transient and of too low affinity to resist pull-down experiments. Thirdly, interactions between UBL3 and MARCH1 or MHC II may require the preservation of membrane integrity, which is disrupted upon membrane solubilization. Finally, the proportion of MHC II molecules ubiquitinated at any time is small^4,33,34^, so the number of interactions occurring between UBL3 and MHC II or MARCH1 may likewise be very small. We resorted to confocal microscopy to gain further insights into the role of membrane anchoring of UBL3 in MHC II ubiquitination. While mutant Myc-UBL3_C114S_ was distributed throughout the cytosol as expected, its WT counterpart appeared concentrated in membrane structures that excluded MHC II (Fig. 3c). This observation suggests that MHC II molecules arriving at membrane sites enriched in prenylated UBL3 undergo ubiquitination, internalization, and degradation and are therefore reduced in number at those locations. Indeed, flow cytometry analysis showed that transduction of Myc-UBL3_WT_ into Δ*Ubl3* MutuDCs reduced the surface expression of MHC II and CD86 to levels similar to those in control *hBim* MutuDCs, whereas transduction of mutant Myc-UBL3_C114S_ did not (Fig. 3d). This supported the notion that prenylated UBL3 was recruited to sites of MHC II and CD86 ubiquitination by MARCH1.

### UBL3 controls MHC II and CD86 surface expression in MARCH1-proficient immune cells

**Fig. 4:**
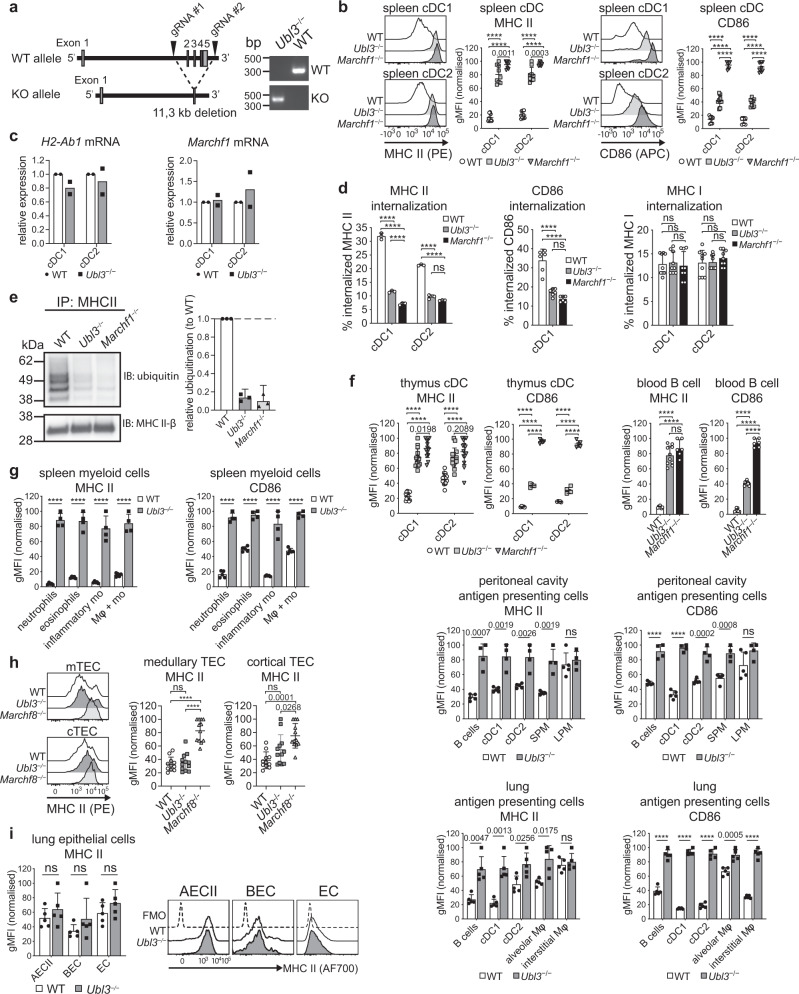
UBL3 regulates MHC II and CD86. **a** *Ubl3*^−/−^ mice. **b** Spleen cDC surface MHC II and CD86 by flow cytometry. Representative histograms and graphs showing gMFI relative to highest signal, bars mean ± SD, symbols  individual mice (*n* = 9, three independent experiments). One-way ANOVA with Bonferroni’s test. **c** Quantitative real-time PCR of *H2-Ab1* and *Marchf1* mRNA in spleen cDCs. Bars mean ± SD relative to WT, *n* = 2 samples, two experiments. **d** Spleen cDCs labeled with FIP-conjugated mAb for MHC II, CD86, or MHC I were incubated for 30 mins at 37°C. Fluorescence was quenched (+Q) and internalization was calculated as described in Methods. CD86 internalization not shown for cDC2 (low expression). Data representative of two independent experiments with similar results (MHC II) or pooled from two (CD86) or three (MHC I) experiments performed in triplicate, mean ± SD, one-way ANOVA with Bonferroni’s test. **e** MHC II was immunoprecipitated (IP) from spleen cDCs using anti-MHC II antibody, analyzed by non-denaturing SDS-PAGE, and immunoblotted (IB) for ubiquitin and MHC II β-chain. Left: representative blot. Right: MHC II ubiquitination relative to WT, bars mean + 95% confidence interval for three biological replicates. **f**–**i** Flow cytometry of surface MHC II and CD86 of **f** professional antigen-presenting cells (thymus: *n* = 14, four experiments (MHC II), *n* = 5, one experiment (CD86); blood: *n* = 7 for WT, 10 for *Ubl3*^−/−^, 6 for *Marchf1*^−/−^, two experiments; peritoneal cavity: *n* = 5 for WT, 4 for *Ubl3*^−/−^, one experiment), **g** spleen myeloid cells (*n* = 5 for WT, 4 for *Ubl3*^−/−^, one experiment), **h** TEC (*n* = 11 for WT, 12 for *Ubl3*^−/−^, 12 for *Marchf8*^−/−^, four experiments), and **i** lung epithelial cells (*n* = 5, one experiment). Graphs show gMFI relative to highest gMFI, bars mean ± SD, symbols represent individual mice. One-way ANOVA with Bonferroni’s test, unpaired *t* test (two-sided) with Holm–Sidak correction. *SPM* small peritoneal macrophages, *LPM* large peritoneal macrophages, *Mφ* macrophages, *mo* monocytes, *AECII* type II lung alveolar epithelial cells, *BEC* bronchial epithelial cells, *EC* lung endothelial cells. *p* values above bars, with ****p < 0.0001, ns (not significant) *p* > 0.05.

Having shown UBL3 is a co-factor for MARCH1 ubiquitination in MutuDCs, we reasoned that mice lacking UBL3 would phenocopy most or all of the effects described in MARCH1-deficient mice. To test this, we generated *Ubl3* knockout mice using CRISPR-Cas9 with two gRNAs flanking *Ubl3* exons 2–5, where the intervening genomic DNA sequence was excised, resulting in a null allele (Fig. 4a). *Ubl3*^−/−^ mice were viable and reproduced normally.

A previous study reported that UBL3 binds to membrane proteins and this post-translational modification regulates the inclusion of the modified proteins in small extracellular vesicles (sEV), membranous structures that are generated in multivesicular bodies of cells and secreted to the extracellular environment^35^. Mice deficient in UBL3 reportedly have reduced protein content in blood sEV^27^. In contrast to this study, we did not observe reduced protein content in sEV purified from the serum of our *Ubl3*^−/−^ mice (Supplementary Fig. 3).

Next, we analyzed by flow cytometry the role of UBL3 in the regulation of MHC II and CD86 expression in the two types of splenic cDCs, cDC1, and cDC2 (for gating see Supplementary Fig. 4). Both surface proteins were more highly expressed on the two *Ubl3*^−/−^ cell types than on their WT counterparts. MHC II expression was as high in *Ubl3*^−/−^ cDC as it was in *Marchf1*^−/−^ cDCs, while CD86 was expressed at an intermediate level (Fig. 4b). Transcription of *H2-Ab1* and *Marchf1* was unaffected by the absence of UBL3 (Fig. 4c) Whether UBL3 alters MHC II and CD86 internalization from the plasma membrane was investigated using a DNA-based fluorescence internalization probe (FIP) that enables the relative quantification of internalized surface molecules to be measured by flow cytometry^36,37^. Briefly, spleen cDC1 and cDC2 were incubated with FIP-conjugated mAb specific for each surface molecule. After incubation at 37 °C to allow internalization of receptor-mAb complexes, a complementary quencher-DNA probe (Q) was added to eliminate the fluorescent signal from complexes remaining on the cell surface. Therefore, the intensity of the remaining fluorescent signal is directly proportional to the amount of internalized molecules. *Ubl3*^−/−^ cDC1 and cDC2 displayed significant reductions in internalized surface MHC II and CD86, similar to *Marchf1*^−/−^ cDCs^11^ (Fig. 4d). This was specific for these substrates, not caused by increased turnover of plasma membrane overall, as MHC I internalization was not altered in *Ubl3*^−/−^ cDCs (Fig. 4d). We then examined how UBL3 impacts MARCH-mediated ubiquitination of MHC II. To do this, MHC II was immunoprecipitated from WT, *Ubl3*^−/−^ and *Marchf1*^−/−^ spleen cDCs, and MHC II-associated ubiquitin was assessed by western blotting. As expected, MHC II immunoprecipitated from WT spleen cDCs was associated with a poly-Ub chain that was no longer detected in the absence of MARCH1^2,3^. Notably, the lack of UBL3 also resulted in a loss of MHC II-associated Ub (Fig. 4e). Together, these results indicate that UBL3 regulates the surface expression of MARCH substrates in primary cDCs by enhancing MARCH1-mediated ubiquitination and altering MARCH1 substrate plasma membrane internalization. UBL3 played a similar role in cDCs of the thymus, lungs, and peritoneal cavity and also in other professional antigen-presenting cells, namely B cells, and macrophages (Fig. 4f). Furthermore, we have recently demonstrated that neutrophils, eosinophils, and monocytes, which have generally been considered to lack antigen-presenting function, in fact, express MHC II, but at negligible levels on the cell surface owing to MARCH1 ubiquitination^10^. All these cell types expressed high levels of MHC II and CD86 in *Ubl3*^−/−^ mice (Fig. 4g). Constitutive expression of UBL3 in neutrophils, eosinophils, and monocytes supports the notion that these are atypical antigen-presenting cells, equipped with conserved machinery for antigen presentation, potentially capable of presenting antigen to T cells under certain conditions^38^.

### UBL3 is not required for MARCH8 function in non-hematopoietic cells

In contrast to hematopoietic immune cells, MARCH1 is not expressed in TEC, which instead use the closely related ligase MARCH8 to regulate MHC II expression (Fig. 4h)^5,6^. Surprisingly, MHC II was not altered in *Ubl3*^−/−^ medullary or cortical TEC (Fig. 4h), even though medullary TEC express UBL3 at levels comparable to DCs or B cells according to the Immunological Genome Project (ImmGen) database^39^. Similarly, we found no changes in MHC II surface expression on type II lung alveolar epithelial cells (AECII), bronchial epithelial cells (BEC) or endothelial cells (EC) of *Ubl3*^−/−^ mice compared to WT, even though these cell types also employ MARCH8 to regulate MHC II expression^10^ (Fig. 4i). These results imply that the *Ubl3* deletion does not cause overall altered membrane protein expression in vivo and confirm UBL3 as a specific co-factor for MARCH1 ubiquitination.

### UBL3 controls regulatory T-cell development in the thymus

**Fig. 5:**
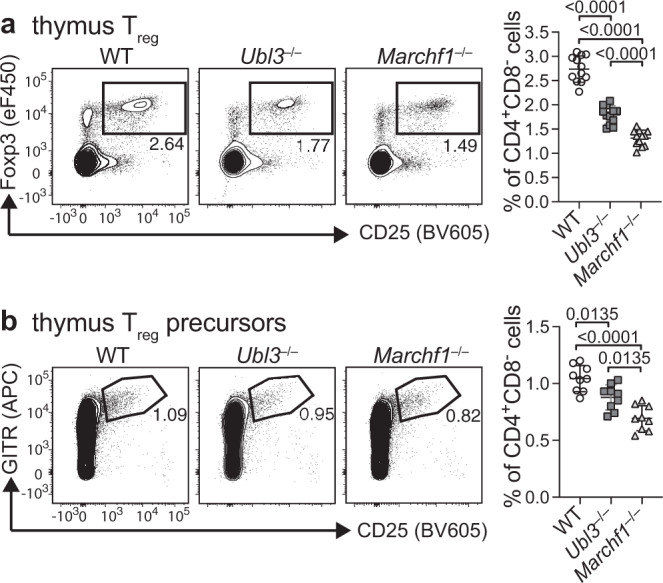
UBL3 is required for normal thymic T_reg_ development. Quantification of **a** Foxp3^+^CD25^+^ T_reg_ or **b** Foxp3^−^CD25^+^GITR^+^ T_reg_ precursor frequencies in thymus. Representative plots are shown on the left. Graphs on the right show pooled data from **a** *n* = 12 mice examined over four experiments and **b** *n* = 9 mice examined over three experiments, with each symbol representing an individual mouse and bars designating mean ± SD. *P* values shown above bars, one-way ANOVA with Bonferroni’s multiple comparisons test.

The physiological role of UBL3 in immunity was examined in two different scenarios in which MHC II regulation critically determines T-cell fate: regulatory T-cell (T_reg_) development and CD4^+^ T-cell priming. MHC II ubiquitination by MARCH1 in DCs is necessary for normal thymic T_reg_ development^9,12,40^, so *Marchf1*^−/−^ mice or mice expressing an MHC II molecule that cannot be ubiquitinated have reduced frequency of Foxp3^+^CD25^+^ T_reg_ in the thymus compared to WT mice (Fig. 5a). *Ubl3*^−/−^ mice also displayed a reduced proportion of thymic T_reg_, intermediate between those of WT and *Marchf1*^−/−^ mice. Similarly, Foxp3^-^CD25^+^GITR^+^ T_reg_ precursors^41^ were significantly reduced in *Ubl3*^−/−^ thymi (Fig. 5b), indicating UBL3 function is important in early T_reg_ development.

### UBL3 is required for cDC homeostasis and antigen presentation in vivo

**Fig. 6:**
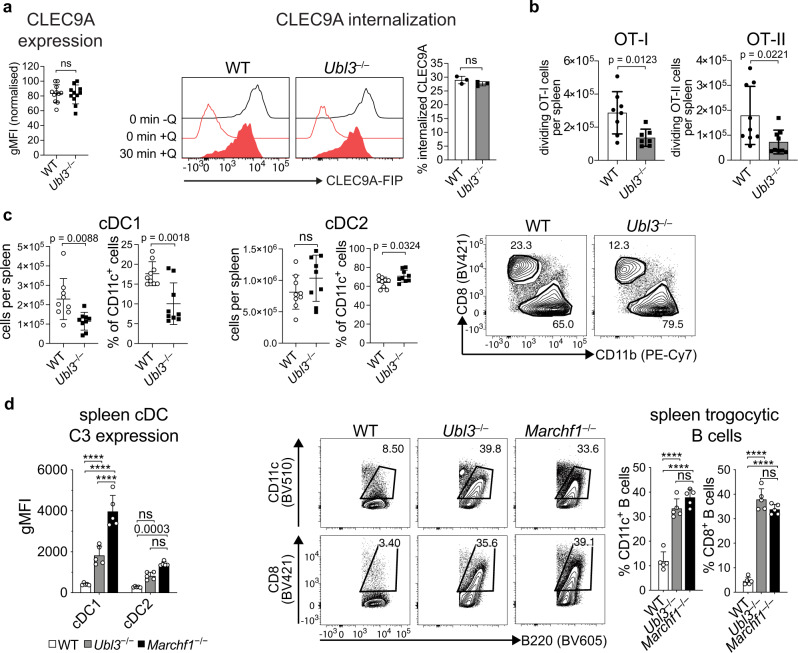
Lack of UBL3 impairs DC-targeted antigen presentation in vivo. **a** Left: spleen cDC1 expression of CLEC9A from wild type (WT) or *Ubl3*^−/−^ mice by flow cytometry, *n* = 11 mice examined over three experiments, bars mean ± SD, symbols represent individual mice, ns (not significant) *p* > 0.05, unpaired *t* test (two-sided). Right: Spleen cDCs from WT or *Ubl3*^−/−^ mice were labeled with FIP-conjugated anti-CLEC9A mAb. After 30 mins at 37°C, quencher (Q) was added and percentage internalization was calculated as described in Methods. Histograms show representative CLEC9A-FIP signal. Right: quantification of internalization from one experiment performed in triplicate, with bars mean ± SD, ns (not significant) *p* > 0.05, unpaired *t* test (two-sided). **b** In vivo antigen presentation assay. Purified CellTrace Violet (CTV)-labeled OT-I and OT-II cells were adoptively transferred into WT and *Ubl3*^−/−^ mice. 24 hours later, mice were injected with 0.2 µg anti-CLEC9A mAb-targeted OVA and spleens harvested after 64 hours. Antigen presentation capacity was assessed by flow cytometric analysis of CTV-dilution by OT-I and OT-II proliferation. Data from *n* = 9 (OT-I) and *n* = 8 for WT, 7 for *Ubl3*^−/−^ (OT-II) mice, two experiments, bars mean ± SD, symbols represent individual mice, ns (not significant) *p* > 0.05, unpaired *t* test (two-sided). **c** Quantification of spleen cDC1 and cDC2 from WT or *Ubl3*^−/−^ mice. Data from *n* = 9 mice, three independent experiments, bars mean ± SD, symbols representing individual mice, ns (not significant) *p* > 0.05, unpaired *t* test (two-sided). **d** Left: C3 expression on spleen cDCs from WT, *Ubl3*^−/−^ or *Marchf1*^−/−^ mice by flow cytometry. Data from n = 5 from one experiment, bars mean ± SD, symbols represent individual mice. Two-way ANOVA with Bonferroni’s test. Right: Quantification of trogocytic B cells gated on B220^+^CD19^+^ cells in Nycodenz-enriched splenocytes from WT, *Ubl3*^−/−^ or *Marchf1*^−/−^ mice. Representative dot plots and data from one experiment with *n* = 5, bars mean ± SD, and symbols representing individual mice. Significant *p* values shown above bars, with ****p < 0.0001, ns (not significant) *p* > 0.05, one-way ANOVA with Bonferroni’s test.

Next, we examined whether mice lacking UBL3 display similar defects in antigen presentation and T-cell activation as those described in other mutant mice defective in MHC II ubiquitination. WT or *Ubl3*^−/−^ mice were injected intravenously with CellTrace Violet-labeled IA^b^-OVA_323-339_-specific OT-II x Ly5.1 cells or K^b^-OVA_257-265_-specific OT-I x Ly5.1 cells. The following day, mice were immunized intravenously with rat anti-CLEC9A mAb genetically fused to the model antigen ovalbumin (OVA). This mAb delivers OVA specifically to cDC1 that express high levels of C-type lectin CLEC9A^42^. CLEC9A expression was unchanged in *Ubl3*^*−/−*^ cDCs and its internalization was similar for *Ubl3*^*−/−*^ and WT cDC1 (Fig. 6a). Three days after immunization, analysis by flow cytometry revealed significantly reduced proliferation of OVA-specific OT-II, and OT-I cells in the spleens of *Ubl3*^−/−^ mice (Fig. 6b), recapitulating observations made in mice that cannot ubiquitinate MHC II molecules^14^. This was not the case for all antigen forms, as the presentation of untargeted OVA protein was unaffected by *Ubl3* deletion (Supplementary Fig. 5).

One reason why mice that cannot ubiquitinate MHC II molecules are defective in antigen presentation is because they have reduced cDC numbers^10,13,14^. This was also the case in *Ubl3*^−/−^ mice, with the Clec9A expressing cDC1 population particularly affected (Fig. 6c). The number of thymic cDC1 and splenic plasmacytoid dendritic cells (pDC) was normal (Supplementary Fig. 6b). The cause of the reduction in splenic cDC1 in mice defective in MHC II ubiquitination is the retention of MHC II molecules covalently bound to complement component 3 fragment, C3dg, on the cell surface^13^. This triggers excessive trogocytic acquisition of cDC1 plasma membrane by marginal zone B cells, which express the C3dg receptor, complement receptor 2, leading to cDC1 elimination and accumulation of marginal zone B cells displaying cDC1 membrane proteins on their surface^13^. *Ubl3*^−/−^ mice also displayed high levels of C3dg on cDC1 and contained large numbers of trogocytic B cells (Fig. 6d).

### Impaired antigen presentation by cDCs lacking UBL3 is not due to processing defects

**Fig. 7:**
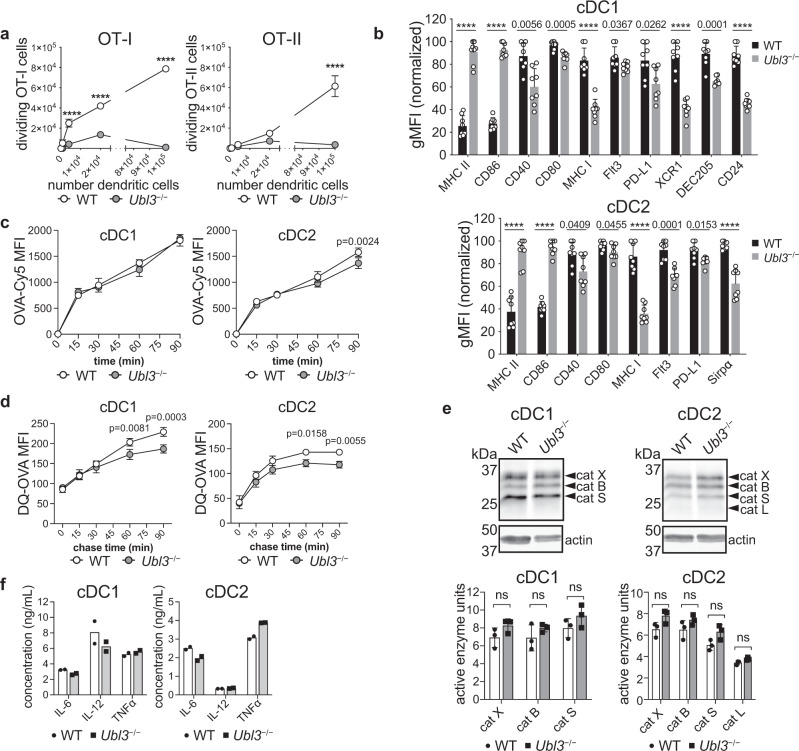
Lack of UBL3 alters cDC function and phenotype. **a** Ex vivo antigen presentation assay. WT and *Ubl3*^−/−^ mice were injected with 1 µg of anti-CLEC9A-OVA mAb. Indicated numbers of spleen cDC1 and cDC2 were purified and co-cultured with OT-I or OT-II cells, and divided OT-I or OT-II cells enumerated by flow cytometry. Data with *n* = 3, representative of two experiments, mean ± SD, ****p < 0.0001, two-way ANOVA with Bonferroni’s test. **b** Flow cytometry analysis of relative cell surface marker expression of spleen cDC1 or cDC2 isolated from WT and *Ubl3*^−/−^ mice, with *n* = 8 mice, two independent experiments, symbols represent individual mice, bars mean ± SD, ****p < 0.0001, unpaired *t* test (two-sided) with Holm–Sidak adjustment. **c** OVA-Cy5 uptake assay. Purified spleen cDC1 or cDC2 from WT or *Ubl3*^−/−^ mice were incubated with 50 µg/ml OVA-Cy5 for the indicated times, and washed before flow cytometry analysis. Graphs show mean ± SD, with data from one experiment performed in triplicate, two-way ANOVA with Bonferroni’s test. **d** Proteolysis assay. Purified spleen cDC1 or cDC2 from WT or *Ubl3*^−/−^ mice were pulsed with DQ-OVA for 15 min, washed twice and DQ-OVA signal was measured by flow cytometry at different chase time points. Graphs show mean ± SD, with data pooled from two independent experiments performed in triplicate, two-way ANOVA with Bonferroni’s test. **e** Quantification of cathepsin (cat) activity in spleen cDCs. Top: representative gel indicating active cathepsin X/B/S/L, and actin immunoblot. Bottom: relative protease activity normalized to actin, with bars showing mean ± SEM of one experiment with three biological replicates. Four spleens were pooled for each biological replicate, ns not significant, unpaired *t* test (two-sided). **f** Cytokine expression of purified *Ubl3*^−/−^ and wild-type spleen cDC1 and cDC2 stimulated with CpG, IFN-γ, and GM-CSF. Bars display mean ± SD of cells stimulated in duplicate, using purified cDCs pooled from eight mice, representative of two independent experiments.

While a reduction in cDC1 numbers likely contributes to reduced antigen presentation in *Ubl3*^−/−^ mice, cDC-intrinsic defects were also possible. To test this, an ex vivo antigen presentation assay was performed. WT or *Ubl3*^−/−^ mice were injected with anti-CLEC9A-OVA mAb. Equivalent numbers of WT and *Ubl3*^−/−^ cDC1 were purified by flow cytometry and co-cultured ex vivo with OT-I and OT-II cells. *Ubl3*^−/−^ cDC1 showed reduced capacity to stimulate both OT-I and OT-II proliferation ex vivo (Fig. 7a). We analyzed the surface expression of characteristic cDC markers in both cDC1 and cDC2 and observed significant decreases in expression of CD40, CD80, MHC I, programmed death-ligand 1 (PD-L1), X-C motif chemokine receptor 1 (XCR1), dendritic and epithelial cells, 205 kDa (DEC205) and CD24 in cDC1, and PD-L1, FMS-like tyrosine kinase 3 (FLT3) and signal-regulatory protein α (Sirpα) in cDC2 (Fig. 7b, Supplementary Fig. 7a). Therefore, *Ubl3*^−/−^ DCs exhibit an altered phenotype extending to markers other than MHC II and CD86. In contrast to DCs, follicular B cells expressed normal levels of all surface molecules we examined except MHC II and CD86 (Supplementary Fig. 7b, c). These results again recapitulate observations made in mice defective in MHC II ubiquitination^14^.

Next, we assessed antigen processing of spleen cDCs. We hypothesized that if the lack of UBL3 impacted endosomal dynamics and function, it may alter antigen internalization or degradation by endosomal cathepsins. However, no major difference was detected for OVA uptake by WT or *Ubl3*^−/−^ cDCs (Fig. 7c). Processing of double quenched (DQ)-OVA was marginally reduced for cDC2, however, no striking differences were observed that sufficiently explained the reduction in antigen presentation (Fig. 7d). Cathepsin protease activity was unaffected by the absence of UBL3 in cDC1 or cDC2 as profiled using an active site fluorescent probe that covalently binds to active cathepsin B, L, S, and X^43^ (Fig. 7e).

Finally, we measured cytokine secretion upon CpG encounter as a surrogate assessment of multiple aspects of intracellular trafficking and endosomal fitness, involving CpG internalization via DEC205^44^, its binding, and signaling through endosomal TLR9^45^, and cytokine release by exocytosis. *Ubl3*^−/−^ cDCs showed similar production of IL-6, IL-12 and TNFα compared to WT cDCs upon stimulation with CpG (Fig. 7f). This result argues against overt disruption of endosomal trafficking and integrity in cells deficient in UBL3.

The conclusion of our experiments addressing functional properties of UBL3-deficient cDCs is that this mutation recapitulates the main defects caused by impaired MARCH1-mediated ubiquitination, with little evidence for other alterations attributable to defective endosomal functions.

### The function of UBL3 is conserved in mouse and human immune cells

**Fig. 8:**
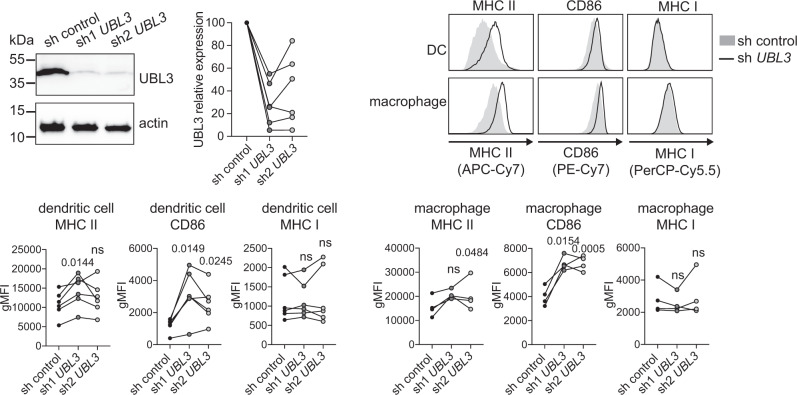
UBL3 function is conserved in humans. Human CD14^+^ monocytes were infected with non-targeting control shRNA or shRNA against *UBL3* (sh1 *UBL3* or sh2 *UBL3*) and analyzed after five days in culture. Top left: Cell lysates were probed with antibodies against UBL3 (LS-C661402) and actin, and quantification of gene silencing was determined by UBL3 signal intensity. Immunoblot depicts a scenario of approximately 95% UBL3 knockdown. Data from *n* = 6 donor samples examined in a single experiment, with symbols connected with a line representing individual donors. Top right: Surface MHC II, CD86, and MHC I on monocyte-derived DCs (CD16^−^ CD1a^+^) and monocyte-derived macrophages (CD16^+^ CD1a^−^) were analyzed by flow cytometry. Histograms are representative of analysis from one donor. Bottom: Graphs showing data from *n* = 6 (DCs) or *n* = 4 (macrophages) donor samples examined in a single experiment, with symbols connected with a line representing individual donors, *p* values shown above points, ns not significant, repeated measures one-way ANOVA.

To determine if UBL3 also plays a role in human cells, its expression was silenced in human monocytes using lentivirus containing anti-*UBL3* or control shRNA, and monocytes were differentiated into DCs and macrophages (Fig. 8). *UBL3* silencing significantly increased MHC II levels in both monocyte-derived DCs and macrophages with at least one of two anti-*UBL3* shRNA tested, compared with control, while CD86 levels were increased with both anti-*UBL3* shRNA, whereas MHC I levels were unaffected. Therefore, UBL3 regulates MHC II and CD86 in murine and human primary antigen-presenting cells.

## Discussion

We have identified UBL3 as a new and critical component of the MARCH ubiquitination machinery. Human and mouse cells that lack UBL3 expression show impaired MHC II ubiquitination, triggering a cascade of phenotypic and functional effects that closely recapitulate those caused by other mutations that impair MHC II ubiquitination.

UBL3 is a member of the ubiquitin-like protein (UBL) family, possessing the typical beta-grasp fold^46^, without obvious sequence homology to ubiquitin itself^30^. Other members of this family include NEDD8, SUMO, ATG8, and ISG15. UBL3 protein is widely expressed in murine tissues, including lung, spleen, ﻿pancreas, kidney, colon, with strong expression in the brain and small intestine^27^. Human UBL3 mRNA is detected in the spleen, thymus, heart, skeletal muscle, pancreas, and prostate, with the highest expression in ﻿the testis, ovary, and brain^31^. In humans, UBL3 gene expression is associated with non-small cell lung cancer^47^, BRCA1/2-negative breast cancer^48^, and cervical carcinoma^49^. Prior to our study, the function of UBL3 was only examined in vitro^22,46^, in-plant cells^21,30^, and in one study describing UBL3 as a protein modifier required for protein sorting into sEV originating in multivesicular bodies^27^. This activity was reportedly mediated by the covalent binding of UBL3 to other proteins, acting as a post-translational modifier analogous to ubiquitin and other UBL^30^. We obtained evidence of possible UBL3 binding to other proteins, but we were unable to confirm its role in sEV formation in vivo, perhaps owing to differences in mouse strains used, protocols for sEV purification, or analytical tools. Our results also showed that the absence of UBL3 did not affect the abundance of active endosomal proteases, the degradation of endocytosed antigens, the processing and function of TLR9, and the MHC II chaperone Ii, or the acquisition of peptide cargo by newly synthesized MHC II molecules. This suggested that the architecture of the endocytic route in UBL3-deficient cells was not as dramatically altered as might be expected if this mutation had disrupted the dynamics of the formation of multivesicular bodies.

The UBL3 activity for which we obtained unequivocal evidence was in MARCH1-mediated ubiquitination. Cell lines or primary cells known to employ MARCH1 for MHC II and CD86 ubiquitination^2,3,10^ expressed high levels of these two receptors in the absence of UBL3, phenocopying the effect of MARCH1 deficiency or, in the case of MHC II, the replacement of its β chain with a mutant form that cannot be ubiquitinated^14,40^. Furthermore, UBL3-deficient cells can generate ubiquitinated MHC II molecules. Interestingly, cells that utilize MARCH8 to ubiquitinate MHC II^5,6^ displayed normal levels of this surface receptor in UBL3-deficient mice, proving the absence of UBL3 was not sufficient to alter membrane protein expression. This raises the question of how MARCH8 and MARCH1 differ in their requirements for UBL3, given their RING-CH domains are highly similar. There may be putative adaptor proteins in epithelial cells that recruit ubiquitination machinery similar to UBL3. In any case, the corollary of these observations is that UBL3 is a specific MARCH1 co-factor.

How does UBL3 assist MARCH1 ubiquitination? A mutation that prevented its prenylation (Cys114) impaired function, suggesting UBL3 activity requires recruitment to membrane sites of MARCH1 ubiquitination. *Arabidopsis thaliana* UBL3 isoforms are also anchored to the membrane by prenylation^30^ and bind E2 enzymes *in planta*^21^. Both *A. thaliana* and human UBL3 bind group VI family E2s, ﻿related to the mammalian UBE2D E2 family, and tether them to membranes^21^. This interaction has been mapped to the “backsite” of group VI E2s^22^, where it does not obstruct the catalytic site containing the Ub-binding Cys. An attractive model for UBL3 activity in MARCH1-mediated ubiquitination could therefore include its recruitment of E2 enzymes to membranes, interacting with both an ﻿E2 and MARCH1 to orient them for efficient Ub transfer to substrates (Supplementary Fig. 10). We were unable to pull-down MARCH1 in UBL3 immunoprecipitation experiments, but this was probably due to the low levels of endogenous MARCH1^2,50^, the transient nature of this interaction, and the disruptive effect of membrane solubilization required for immunoprecipitation. The formation of complexes involving MARCH1, E2, and possibly other adaptors that promote ubiquitination of membrane substrates is not without precedent. For example, MHC II ubiquitination can also involve *Salmonella* effector protein SteD, which acts as an adaptor for host E3 ligase WW domain-containing protein 2 (WWP2) and co-factor transmembrane protein 127 (TMEM127)^51^. These observations and our own lead us to propose a model where UBL3 forms complexes with MARCH1, E2 ligase(s), and possibly other co-factors to ubiquitinate MHC II and CD86. It is also conceivable that other E3 ligases cooperate with UBL3 to ubiquitinate membrane substrates (Supplementary Fig. 10). Confirmation of this model awaits further investigation.

A clear conclusion of our study is that UBL3 plays an important role in immunity. Mice that lack UBL3 display multiple immune defects that can be attributed primarily to defective MARCH1-mediated MHC II ubiquitination, as they are equivalent to those we and others have observed in mice that only carry a point mutation in MHC II that prevents its ubiquitination^9,14,15,40^. MHC II is an abundant protein in antigen-presenting cells. Its endocytosis, recycling, and turnover rates are dramatically altered in the absence of ubiquitination, causing its “overcrowding” on the plasma membrane, which has secondary effects on the expression of other membrane proteins by mechanisms that are not fully understood but may include disruption of lipid rafts and the tetraspanin network^12^. This explains the altered expression of multiple membrane receptors in mice that lack MARCH1^13^, cannot ubiquitinate MHC II^11,12,14^ or, as we show here, do not express UBL3. Another effect of impaired MHC II ubiquitination is the accumulation of MHC II molecules covalently bound to C3dg on the surface of antigen-presenting cells^13^, also observed in *Ubl3*^−/−^ mice.

The cell-intrinsic effects of altered MHC II ubiquitination that we have described have important downstream consequences on immune cell numbers and function and, ultimately, immunity. First, altered expression of MHC I and II, and, perhaps other surface receptors, contributed to cell-intrinsic defects in cDC antigen presentation. Second, high MHC II expression by thymic cDCs resulted in impaired T_reg_ selection in the thymus of *Ubl3*^−/−^ mice, as it did in *Marchf1*^−/−^ mice^9,12^ and mice that express an MHC II molecule that cannot be ubiquitinated^40^. Third, the accumulation of MHC II-C3dg complexes on splenic cDC1 made them highly susceptible to trogocytosis by marginal zone B cells^13^, causing a reduction in cDC1 numbers, a phenomenon also observed in *Marchf1*^−/−^ mice and mice that cannot ubiquitinate MHC II^13,14^. The similarities of effects of UBL3 and MARCH1 deficiencies on complex immune functions support the notion that the primary role of UBL3 is primarily to assist MARCH1 ubiquitination, though naturally, we cannot discard its involvement in other, yet undescribed activities. An attractive possibility is cooperation with other members of the MARCH family. We did not observe altered MARCH8 activity in *Ubl3*^−/−^ mice, but this does not discard its potential involvement with other mammalian or viral members of the family or even different types of E3 ligases. Given the emerging roles of MARCH E3 ligases in viral immunity^16^, whether UBL3 is involved in infection or anti-viral defense are interesting questions that remain to be explored.

In conclusion, through unbiased genome-wide screening, we have discovered the small UBL3 as a new molecule critical for MARCH-mediated ubiquitination. Our findings highlight UBL3 as a novel regulator of important immunoreceptors including MHC II and CD86 and for cDCs and T_reg_ immunity. Further studies will elucidate how UBL3 interacts with the MARCH1 ubiquitination cascade, and whether it is involved in other ubiquitination pathways.

## Methods

### CRISPR screen

Mouse GeCKOv2 CRISPR knockout pooled library was a gift from Feng Zhang (Addgene #1000000052). The library encodes 67,405 gRNAs, with three different gRNAs targeting 20,611 genes, in addition to miRNAs and non-targeting controls^52^. For the pooled genome-wide CRISPR screen, pooled lentiviral GeCKOv2 half-library A was produced in HEK293T cells and 7.2 × 10^7^ MutuDC1940 were infected by spinoculation for 2 h at 2000 rpm and 32 °C to achieve a functional multiplicity of infection (MOI) of 0.3–0.4 for coverage of >300 over the library. After 4 days, transduced cells were selected with 1 μg/ml puromycin for 5 days. Three independent library replicates were either maintained in culture or stained with PE-conjugated anti-MHC II antibody and sorted (in triplicate) using a Beckton Dickinson Influx cell sorter (BD Biosciences) by gating on the top 5% of the PE channel. Cells were expanded for 10 days and genomic DNA from MHC II^hi^ or unsorted cells (reference library) was isolated using a previously described salt precipitation protocol^53^. For each sample, 100 µg DNA was used to amplify the gRNA cassette by two-step PCR described previously^54^ using Hot Start Taq Polymerase (New England Biolabs), however in the second PCR we incorporated the 8 bp barcode into the reverse as well as a forward primer. Barcoded amplicons were pooled and purified using Nucleo Mag NGS beads (Macherey-Nagel), then sequenced using a NextSeq 500 (Illumina). CRISPR/Cas9 library data processing and differential representation analysis were performed using an established bioinformatics pipeline^55^. Negative binomial linear models of gRNA count data incorporated blocking terms to account for any batch effects introduced by independent library infections. Significant enrichment of gRNAs in experimental samples relative to controls was determined by false discovery rate adjustment of *p* values. Enriched gRNAs with a fold change ratio of >2 and false discovery rate <0.01 were considered hits.

### Generation of Δ*Ubl3* MutuDC cell lines

MutuDC1940 cells were a gift from Hans Acha-Orbea (University of Lausanne, Switzerland)^20^. Cas9-expressing MutuDCs were generated by lentiviral transduction of lentiCas9-Blast (Addgene plasmid # 52962), gifted by Feng Zhang, and selection of transduced cells with 10 µg/ml blasticidin for 3 days. To generate CRISPR knockout cell lines, ﻿sgRNAs were designed using the Broad Institute GPP sgRNA Designer^56^ and cloned into the FgH1t_UTG vector (Addgene plasmid #70183), gifted by Marco Herold^57^. sgRNA sequences were as follows: *Ubl3* exon 2: 5′-GTGGCCGAGTGTTTAAAGTG-3′; *hBIM* exon 3: 5′-GCCCAAGAGTTGCGGCGTAT-3′; *Marchf1* exon 6: 5′-CTGAGCTCTTGATCCATTGG-3′; *B2m* exon 2: 5′-TCGGCTTCCCATTCTCCGGT-3′. Lentivirally transduced cells were treated with 1 μg/mL doxycycline (Sigma-Aldrich) for 3 days to induce sgRNA transcription, and CFP^+^ cells were sorted a Beckton Dickinson Influx cell sorter (BD Biosciences). Cells were either sorted as a bulk population (referred to as pool), or individual cells sorted into a single well of a 96-well plate to propagate a homogenous cell population (referred to as single-cell clones). Successful genomic cleavage of the target gene was confirmed using Sanger sequencing and inference of CRISPR edits (ICE) analysis platform (Synthego)^23^.

### Radiolabeling and immunoprecipitation of MHC class II

Cells were starved in methionine- and cysteine-free Dulbecco’s Modified Eagle Medium (DMEM) for 30 min at 37 °C and then pulsed with ^35^S-labeled methionine and cysteine (Express Protein Labeling Mix, Perkin Elmer) at 200 μCi/ml for 30 min at 37 °C. After washing with ice-cold complete DMEM with 10% fetal calf serum (FCS; DMEM-10), cells were incubated in this media at 37 °C. At the indicated chase time points, cells were washed in phosphate-buffered saline (PBS) and frozen. Cell pellets were lysed in 0.5% IGEPAL CA-630 (Sigma-Aldrich), 50 mM Tris-HCl (pH 7.4), 5 mM MgCl_2_ with Complete Protease Inhibitor Cocktail (Roche), and nuclei removed by centrifugation at 13,000 × *g* for 10 min. Lysates were pre-cleared twice with normal rabbit serum (NRS; Sigma-Aldrich) and protein G-sepharose beads (Walter and Eliza Hall Institute Antibody Facility), and once with protein G-sepharose alone. MHC II was immunoprecipitated using rabbit antisera against MHC II α chain (JV1) and protein G-sepharose, or compared to NRS alone, and the immunoprecipitates were washed in NET buffer (0.5% IGEPAL CA-630, 50 mM Tris-Cl pH 7.4, 150 mM NaCl, 5 mM ethylenediaminetetraacetic acid (EDTA)) four times. Precipitates were divided into two and a reducing SDS-PAGE sample buffer was added to each; one was heated at 95 °C for 5 min and the other left at room temperature. Proteins were separated on NuPAGE 4–12% Bis-Tris precast gels (Life Technologies) before transferring onto polyvinylidene difluoride (PVDF) membranes using the iBlot2 system (Life Technologies). Membranes were dried and exposed to a storage phosphor screen (GE Healthcare) and imaged on a Typhoon imager (GE Healthcare).

### UBL3 constructs

To generate cell lines expressing wild type or mutant UBL3 constructs, Myc-tagged wild type or C114S mutant *Ubl3* cDNA was purchased (Biomatik) and cloned into p-MSCV-IRES-eGFP II (Addgene plasmid #52107), which was a gift from Dario Vignali. Single-cell cloned Δ*Ubl3* MutuDCs were transduced with retroviral supernatant, and GFP^high^ expressing cells were sorted with a Beckton Dickinson Influx cell sorter (BD Biosciences). Expression of Myc-tagged UBL3 constructs was confirmed by western blotting using antibodies against UBL3 (ab113820, Abcam), Myc-tag (71D10, Cell Signaling Technologies), and actin (20-33, Sigma) antibodies. For analysis of surface markers, cells were stained with antibodies against MHC II (M5/114) and CD86 (GL-1) and analyzed by flow cytometry on the LSRFortessa (BD Biosciences). For full details of antibodies, see Supplementary Tables 3, 4.

### Immunoprecipitation

MutuDCs or spleen cDCs were lysed on ice in lysis buffer (20 mM Tris pH 7.5, 150 mM NaCl, 1 mM EDTA, 1% v/v Triton X-100) supplemented with 10 mm *N*-ethylmaleimide (Sigma-Aldrich) and cOmplete Mini EDTA-free protease inhibitor cocktail (Roche). Post-nuclear supernatants were pre-cleared by incubation with uncoupled protein G-sepharose beads (Walter and Eliza Hall Institute Antibody Facility). MHC II was immunoprecipitated with anti-MHC II mAb (M5/114) coupled to protein G-sepharose beads (Walter and Eliza Hall Institute Antibody Facility), and Myc-tag was immunoprecipitated with anti-c-Myc-tag (9E10) affinity gel (BioLegend). Samples were eluted by denaturation in non-reducing sample buffer at 95 °C and analyzed by western blot analysis.

### PLA and confocal microscopy

For confocal microscopy, MutuDCs were attached to coverslips treated with anti-MHC II antibody (10 μg/mL, clone N22). Cells were fixed with 4% paraformaldehyde/PBS, permeabilized with 0.3% Triton X-100 in PBS and blocked with 0.1% Triton X-100, 10% FCS in PBS, and stained with mouse mAb against Myc-tag (clone 9E10, Cell Signaling Technology), biotinylated rat mAb against MHC II (clone M5/114, WEHI antibody facility), goat anti-mouse Alexa Fluor 594 (Thermo Fisher), Alexa Fluor 647-conjugated avidin (made in house), and 0.5 μg/ml DAPI (Thermo Fisher). For full details of antibodies, see Supplementary Table 5. Coverslips were mounted in SlowFade Diamond (Thermo Fisher) and analyzed on a Leica SP8 Confocal microscope (Biological Optical Microscopy Platform, University of Melbourne. For PLA, MutuDCs were grown overnight on glass coverslips treated with anti-MHC II antibody (10 μg/mL, clone N22) and stained with plasma membrane CytoPainter (Abcam). After extensive washes, cells were fixed with 4% paraformaldehyde in PBS and permeabilized with 0.3% Triton X-100/PBS. Cells were blocked with blocking buffer and stained with rabbit antiserum against MHC class II (JV2) and mouse mAb against Myc-tag (clone 9E10) diluted in antibody diluent with Duolink In Situ PLA Probe Kit (Sigma-Aldrich) as instructed. PLA was performed using the Duolink Mouse/Rabbit Far Red Kit (Sigma-Aldrich) according to the manufacturer’s instructions. After staining with Hoechst 33342, slides were mounted in 90% glycerol, 20 mM Tris-Cl pH 8, 0.2 M DABCO, and imaged using a Zeiss LSM780 confocal microscope. Imaris (version 9.1.2) was used to enumerate the number of PLA spots. Each cell was segregated using the ‘Surfaces’ function, then the PLA spots were enumerated within each region using the ‘Spots’ function, using consistent settings for each image. Two distinct regions (containing > 40 cells) of each slide were enumerated for each sample, and statistical analysis was performed with unpaired Welch’s *t* test. (Prism 6, GraphPad).

### Mice

*Ubl3* mice were generated by the MAGEC laboratory (WEHI) as previously described^58^ on a C57BL/6 J background. To generate *Ubl3*^−/−^ mice, 11,278 bp of genomic sequence including *Ubl3* exons 2–5 was targeted for deletion. 20 ng/μl of Cas9 mRNA and 10 ng/μl of sgRNA (sequences sgRNA1: ggcctcgaccatccgagtaa, sgRNA2: gcctgcaagcaaaccgttta) were injected into the cytoplasm of fertilized one-cell stage embryos. Twenty-four hours later, two-cell stage embryos were transferred into oviducts of pseudo-pregnant female mice. Viable offspring were genotyped by next-generation sequencing. C57BL/6 (H-2b), *Marchf1*^−/− 2^, *Marchf8*^−/− 5^, and *Ubl3*^−/−^ mice (this study) were used at 6-12 weeks of age. Experimental and control animals were bred separately, and euthanized by CO_2_ asphyxiation with secondary method cervical dislocation. All mice were bred and maintained in specific pathogen-free conditions at the Melbourne Bioresources Platform at Bio21 Molecular Science and Biotechnology Institute with a 14-hour light/10-hour dark cycle at 18-24 °C and with 40-70% humidity. Experimental procedures were approved by the Animal Ethics Committee of the University of Melbourne (protocol no. 1714375).

### Dendritic cell isolation

Primary spleen or thymic DCs were isolated as previously described^59^. In brief, organs were finely chopped and digested with DNase I (Roche) and collagenase type 3 (Worthington Biochemicals), cell clusters further dispersed by addition of 10 mM EDTA, and light density cells were collected from the upper fractions after density gradient separation in 1.077 g/cm3 Nycodenz (Nycomed Pharma). Cells were washed and either analyzed by flow cytometry (thymus) or subject to further enrichment (spleen) by incubation in a depletion cocktail containing rat anti-mouse mAbs specific for: CD3 (KT3-1.1), Thy1 (T24/31.7), red blood cells (Ter119), B220 (RA36B2), Ly6c/g (RB68C5) (Walter and Eliza Hall Antibody facility). Cells were washed and incubated with BioMag anti-rat IgG-coupled magnetic beads (Qiagen). The cDC-enriched supernatant was recovered by magnetic separation. Absolute cDC numbers were obtained using an internal microsphere counting standard (BD Biosciences). Splenic DCs were stained with mAbs specific for CD11c (N418), CD8 (53-6.7), CD11b (M1/70) (all Biolegend). Thymus cDC preparations were stained with mAbs specific for CD11c (N418), XCR1 (ZET), CD11b (M1/70), B220 (RA36B2), Sirpα (P84) (all Biolegend), and NK1.1 (PK136, BD Biosciences).

### Flow cytometry analysis of primary murine immune cells

Single-cell suspensions were obtained from the spleen, blood, thymus, lung, and peritoneal cavity. Spleen, blood, and peritoneal cavity cells were treated with red cell removal buffer prior to staining. TEC were enriched as previously described^60^. Single thymi were mechanically dispersed followed by sequential digestion with DNase I (Sigma-Aldrich) and liberase (Roche). For analysis of lung cells, mouse right ventricles were perfused with PBS and the lungs were collected, finely chopped, and digested in liberase and DNAse I at 37 °C. Tissue was mechanically disrupted by pipetting, filtered, and treated with red cell removal buffer. For T_reg_ analysis, cells were stained with antibodies against surface markers, fixed and permeabilized with the Transcription Factor Staining Buffer Set (eBioscience), and stained for Foxp3 (FJK-16s, eBioscience) according to the manufacturer’s instructions. For trogocytic B-cell analysis, spleens were chopped and digested with DNase I (Roche) and collagenase type 3 (Worthington Biochemicals), cell clusters further dispersed by addition of 10 mM EDTA, and light density cells collected from the upper fractions after density gradient separation in 1.077 g/cm3 Nycodenz (Nycomed Pharma). For surface marker staining, cells were washed in EDTA-BSS with 2% (v/v) FCS and stained with specific antibodies (see Supplementary Table 3 for a full list of antibodies, suppliers, and dilutions). For C3 staining, cells were incubated in Fc-receptor block (Milteny Biotec), incubated with biotinylated anti-C3 mAb (clone 11H9, Novus Biological), and incubated with DyLight 488-conjugated streptavidin (BioLegend). Stained cells were acquired on the LSRFortessa using FACSDIVA 9.0.1 (BD Biosciences) or CytoFLEX LX (Beckman Coulter) flow cytometer and analyzed with FlowJo software (Tree Star) with the exclusion of cell doublets and dead cells in all cases, identified based on forward and side scatters (FSC and SSC), as well as staining with a fixable viability dye, propidium iodide, or DAPI. Supplementary Fig. 2 shows the gating strategies to identify specific immune cell populations. Statistical analysis was performed with Prism software (Graphpad).

### Real-time quantitative PCR

Total RNA was extracted from DCs isolated from WT or *Ubl3*^−/−^ mice with >90% purity, using the RNeasy Plus Mini Kit (Qiagen). RNA was quantified by UV spectrophotometry (Eppendorf) and reverse transcription of RNA using the SensiFAST cDNA Synthesis Kit (Bioline) was performed according to the manufacturers’ instructions. cDNAs were used as templates for qPCR assay using TaqMan Fast Advanced Master Mix (Life Technologies). Each sample was analyzed in triplicates, and HPRT was used as an internal control. A control without cDNA was also included for each primer set (Thermo Fisher Scientific). Relative gene expression was determined with the ΔΔCq method, with ΔΔCq = (Cq(sample)−(Cq(HPRT)))−(Cq(reference)−(Cq(HPRT))), and fold change = 2^−ΔΔCq^.

### Internalization assay

Fluorescence internalization probe (FIP)-azide (5′-Cy5-TCAGTTCAGGACCCTCGGCT-N3-3′) and quencher (Q; 5′-AGCCGAGGGTCCTGAACTGA-BHQ2-3′) were purchased from Integrated DNA Technologies. To determine receptor internalization, FIP assays were performed as previously described^11,61^. In brief, spleen cDCs were enriched and stained on ice with FIP-Cy5–conjugated mAbs against MHC II (M5/114), CD86 (GL-1), or MHC I (Y3). FIP-labeled cells were incubated in complete RPMI 1640 at 37°C and 10% CO_2_ for 30 mins. Cells were washed, stained with mAbs against CD11c (N418), CD8 (53-6.7), and CD11b (M1/70) (all Biolegend), and resuspended in media containing propidium iodide with or without 1 μM quencher (*Q*). The percentage of internalization was calculated with the equation below, where *Q*_n_ is the gMFI at time *n* after the addition of *Q*, *Q*_0_ is the MFI at time 0 after the addition of *Q*, and *F*_0_ is the gMFI at time 0 without the addition of *Q*.

$$\% {{{{{\rm{internalization}}}}}}=({Q}_{n}\,{-}\,{Q}_{0})/({F}_{0}\,{-}\,{Q}_{0}) {\,}\times 100$$

Data were acquired using a BD LSRFortessa (BD Biosciences) and analyzed with FlowJo software (Tree Star).

### In vivo antigen presentation assay

Single-cell suspensions generated from the lymph nodes of OT-I × Ly5.1 or OT-II × Ly5.1 mice were stained with rat anti-mouse mAbs specific for: CD11b (M1/70), F4/80 (F4/80), red blood cells (TER119), Ly6G/-Ly6C (RB68C5), MHC II (M5/114), CD45R (RA36B2) and CD4 (GK1.5) for OT-I T cells or CD8 (53-6.7) for OT-II cells. Cells were washed and incubated with BioMag anti-rat IgG-coupled magnetic beads (Qiagen). After magnetic depletion, the CD4^+^ T-cell-enriched supernatant was recovered. Cells were washed with PBS 0.1% BSA and labeled with CellTrace Violet. Single-cell suspensions were treated with red cell removal buffer, washed with PBS 0.1% bovine serum albumin (BSA), and labeled with CFSE (Invitrogen). Equal numbers of CTV-labeled OT-II and a control population of carboxyfluorescein succinimidyl ester (CFSE)- labeled splenocytes were pooled and resuspended and injected intravenously. 24 hours later, mice received 0.2 µg anti-CLEC9A-OVA mAb^42^ via intravenous injection. 64 hours after immunization, spleens were harvested, single-cell suspensions generated, and treated with red cell removal buffer. Cells were stained with mAbs specific for CD8 (53-6.7, Biolegend), CD4 (GK1.5; Walter and Eliza Hall Antibody Facility), and Ly5.1 (A20; Biolegend). The number of divided OT-II and OT-I was determined as the number of CD4^+^ or CD8^+^ Ly5.1^+^ cells that had undergone CellTrace Violet dilution. The number of cells was normalized relative to the number of CFSE-labeled splenocytes recovered.

### Ex vivo antigen presentation assay

Mice were intravenously injected with 1 μg of anti-CLEC9A-OVA mAb. 22 hours later, spleen cDC1 and cDC2 were isolated as described above and sorted to purity. OT-I and OT-II cells were purified, labeled with 2 µM CellTrace Violet, and 5 × 10^4^ cells per well were cultured with isolated cDCs (numbers indicated in figures) in U-bottomed 96-well plates for 60 hours (OT-I), or 84 hours (OT-II). The number of divided OT-I and OT-II was determined as the number of CD4^+^ or CD8^+^Ly5.1^+^ cells that had undergone CellTrace Violet dilution.

### Antigen uptake and proteolysis assay

To assess antigen uptake, 5 × 10^4^ sort purified cDC1 or cDC2 were incubated with 50 µg/mL OVA-Cy5 for 0–90 minutes at 37 °C. cDCs were washed twice and the fluorescence was measured by flow cytometry. To measure antigen proteolysis, 2.5 × 10^5^ sort purified cDCs were incubated with 20 µg/mL DQ-OVA (Life Technologies) for 15 min at 37 °C. Cells were washed twice and resuspended in complete medium and chased for up to 90 minutes. At each time point, cells were kept on ice and the fluorescence was measured by flow cytometry. As a control, DCs were pulsed with DQ-OVA for 15 min at 4 °C, washed twice, and analyzed by flow cytometry.

### Cathepsins activity-based probe

Cells were lysed with PBS 0.1% Triton X-100 and total protein concentration was determined by BCA assay. Protein (80 µg) was acidified by adding 10× citrate buffer (final concentration: 50 mM citrate, pH 5.5, 0.5% 3-((3-cholamidopropyl) dimethylammonio)-1-propanesulfonic acid, 0.1% Triton X-100, 4 mM dithiothreitol) or PBS containing 0.1% Triton X-100). BMV109 (pan cysteine cathepsins probe, 1 μM) was added to the acidified cell lysate at 37˚C as previously described^43^. Proteins were resolved by reducing SDS-PAGE and in-gel Cy5 fluorescence was detected using a Typhoon flatbed laser scanner (GE Healthcare). Protease activity (band intensity) was measured by densitometry using ImageJ. Values were normalized to actin (band intensity). Statistical analyses were performed using GraphPad Prism 8.

### Cytokines

Splenic cDC1 and cDC2 were isolated and sorted to purity. In all, 1 × 10^5^ cDCs were cultured in complete medium (RPMI 1640 medium supplemented with 10% heat-inactivated FCS, 1% penicillin/streptomycin, 50 nM 2-ME and 1% GlutaMAX) with 0.5 μM phosphorothioated CpG 1668 (Bioneer), 50 ng/mL interferon-gamma (IFN-γ) (Peprotech) and 20 ng/mL granulocyte–macrophage colony-stimulating factor (Peprotech). Supernatants were collected 18 hours later and stored at −20 °C. Cytokine secretion was determined using the BD Cytometric Bead Array Mouse Inflammation kit according to the manufacturer’s instructions (BD Biosciences).

### Isolation and analysis of human monocytes

Buffy coats from healthy donors were obtained from Etablissement Français du Sang (Paris) in accordance with INSERM ethical guidelines. Peripheral blood mononuclear cells (PBMC) were prepared by centrifugation on a Ficoll gradient (Lymphoprep, Greiner Bio-One). Blood CD14^+^ monocytes were isolated from healthy donors’ PBMC by positive selection using magnetic beads (Miltenyi). Monocytes were 95–98% CD14^+^CD16^−^ as assessed by flow cytometry. Monocytes were cultured in RPMI-Glutamax medium (Gibco) supplemented with antibiotics (penicillin and streptomycin) and 10% FCS in the presence of 100 ng/mL M-CSF (Miltenyi), 5 ng/mL IL-4 (Miltenyi), and 5 ng/mL TNF-α (R&D Biotech). For flow cytometry analysis, cells were stained in PBS containing 0.5% human serum and 2 mM EDTA with antibodies against CD16 (clone 3G8), CD1a (HI149), CD86 (IT2.2), HLA-A,B,C (W6/32) (all BioLegend), and HLA-DR (LN3, eBioscience). Dead cells were identified using LIVE/DEAD fixable Aqua dye (Thermo Fischer Scientific). Cells were fixed using Intracellular Fixation & Permeabilisation Buffer Set (eBioscience) and analyzed on a FACSVerse (BD Biosciences) instrument. Data were analyzed with FlowJo (Tree Star).

### Western blot

For western blot analysis of human monocytes, cells were lysed in radioimmunoprecipitation assay buffer (Thermo Scientific) supplemented with cOmplete Mini EDTA-free protease inhibitor cocktail (Roche), and post-nuclear lysates were resolved by SDS-PAGE using 4–12% Bis-Tris NuPAGE gels (Invitrogen). MutuDCs were lysed in lysis buffer (20 mM Tris pH 7.5, 150 mM NaCl, 1 mM EDTA, 1% v/v Triton X-100) supplemented with cOmplete Mini EDTA-free protease inhibitor cocktail (Roche) and post-nuclear lysates were resolved by SDS-PAGE using 4–12% Bis-Tris Bolt gels (Invitrogen). Proteins were transferred to membranes (Immunoblot PVDF membranes, Bio-Rad). Membranes were stained with primary antibodies against human UBL3 (LSBio, LS‑C661402) or human actin (Millipore, clone C4), followed by horseradish peroxidase (HRP)-conjugated secondary antibodies (Jackson Immunoresearch). For ubiquitin analysis, membranes were probed with HRP-coupled P4D1 (Santa Cruz) or rabbit polyclonal serum generated against I-Ab β-chain (JV2), followed by HRP-conjugated secondary antibodies (Cell Signaling Technology) (for full details see Supplementary Table 4). Signal intensity was measured using a ChemiDoc MP imaging system (Bio-Rad), analyzed with FIJI software, and statistical analysis was performed with Prism (GraphPad).

### shRNA interference

shRNA (all from Sigma) against human UBL3 (sh1: Clone NM_007106.2-591s1c1; sh2: Clone NM_007106.2-757s1c1) or non-targeting control shRNA (MISSION shRNA SHC002) were in the LKO.1-puro vector (Sigma). Gene silencing was performed following an established protocol^62^. In brief, viral particles were produced by transfection of 293FT cells in six-well plates with DNA and TransIT-293 (Mirus Bio) per well: for VSV-G pseudotyped SIVmac VLPs, CMV-VSV-G and pSIV3+; for shRNA vectors, CMV-VSV-G, psPAX2 and LKO1puro-derived shRNA vector. One day after 293FT cell transfection, the culture medium was replaced by a fresh medium. Viral supernatants were harvested 1 day later and filtered. Freshly isolated CD14^+^ monocytes were infected with viral particles containing the control vector or individual shRNA vectors, and cultured as above. Puromycin (InvivoGen) was added after 2 days of culture. On day 5, cells were harvested for analysis.

### Reporting summary

Further information on research design is available in the Nature Research Reporting Summary linked to this article.

## Supplementary information

Supplementary Information

Reporting Summary

## Data Availability

Source data underlying Figs. 1a, b, 2b, 3a, b, d, 4a–i, 5a, b, 6a–d, 7a–e, 8, and Supplementary Figures 2a–c, 3c, 5, 6a, b, and 7c are provided as Source Data file. Proteomics data have been deposited to the ProteomeXchange Consortium via jPOST (accession codes https://repository.jpostdb.org/entry/JPST001444, http://proteomecentral.proteomexchange.org/cgi/GetDataset?ID=PXD030790). The fasta file containing the mouse reference proteome used for the analysis of raw MS data using MaxQuant was retrieved from Uniprot (proteome id UP000000589, downloaded 09-03-2021). Source data are provided with this paper.

## Source data

Source Data
