# Supplementary material for: Ubiquitin-like protein 3 (UBL3) is required for MARCH ubiquitination of major histocompatibility complex class II and CD86
Source: Nat Commun. 2022 Apr 11;13:1934. doi: 10.1038/s41467-022-29524-w (PMC9001657; doi:10.1038/s41467-022-29524-w)
Supplement: Supplementary file 1 — Supplementary Information [file 41467_2022_29524_MOESM1_ESM.pdf]

Supplementary Information

Ubiquitin-like protein 3 (UBL3) is required for MARCH ubiquitination of major histocompatibility complex class II and CD86

Liu et al.

Supplementary figure 1

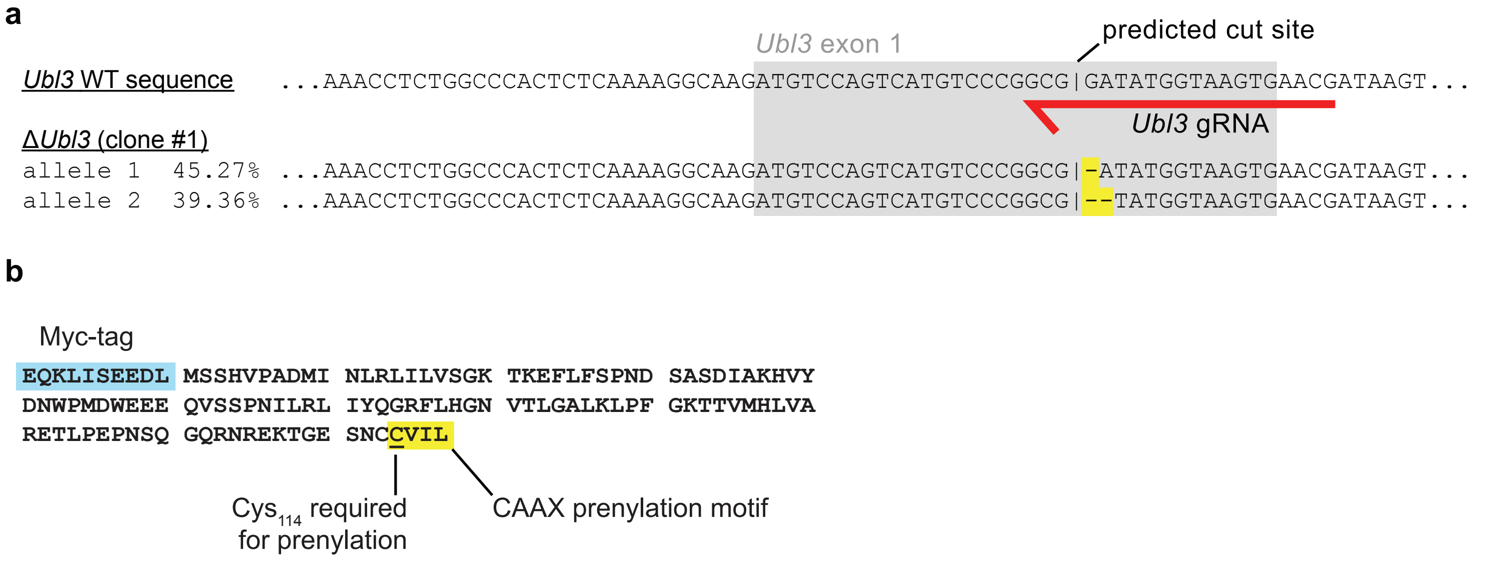

Supplementary figure 1. Details of Δ*Ubl3* MutuDC cell line and UBL3 constructs. (a) Genomic DNA was purified from wild type MutuDC or single cell cloned Δ*Ubl3* MutuDC and the targeted gene region in *Ubl3* exon 1 (grey box) was amplified by PCR. Formation of InDels was analysed using Sanger sequencing and inference of CRISPR edits (ICE) analysis. The WT genomic sequence flanking the targeted site is shown on top, with the 20-nucleotides gRNA target region underlined in red. Sequencing of Δ*Ubl3* MutuDC cells is shown below, including the type of InDel of each predicted allele and their quantitative contribution as calculated by ICE, frameshift mutations are highlighted in yellow. (b) Amino acid sequence of Myc-tagged (blue box) UBL3 constructs used in this study. The prenylation mutant $UBL3_{C114S}$ contains a serine instead of cysteine 114 within the CAAX motif (yellow bow).

Supplementary figure 2

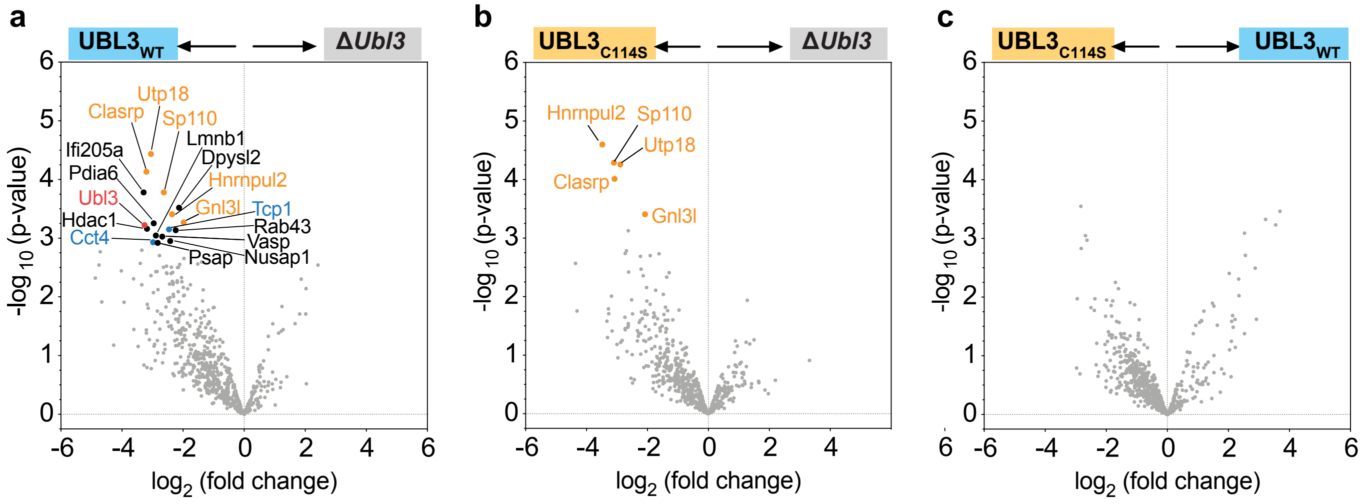

Supplementary figure 2. Volcano plot of mass spectrometry analysis of immunoprecipitated UBL3. Anti-Myc immunoprecipitates from (a) Δ*Ubl3* MutuDC ("Δ*Ubl3*"), or (b-c) Δ*Ubl3* MutuDC retrovirally transduced with (b) Myc-tagged wild type UBL3 ("$UBL3_{WT}$") or (c) $UBL3_{C114S}$ mutant ("$UBL3_{C114S}$") were analysed by mass spectrometry and differential expression analysis was performed using the Bioconductor package limma, with adjustment for multiple testing using the Benjamini-Hochberg false discovery rate (FDR) method. Proteins were considered significantly enriched with an adjusted p-value cut-off <0.05 and log_2 fold change >1 (see methods). 1329 proteins were detected, with 637 proteins analysed by LFQ. Black = significantly enriched proteins, red = UBL3, yellow = proteins enriched in both $UBL3_{WT}$ vs Δ***Ubl3*** and $UBL3_{C114S}$vs Δ*Ubl3*, and blue = proteins involved in tubulin folding.

Supplementary figure 3

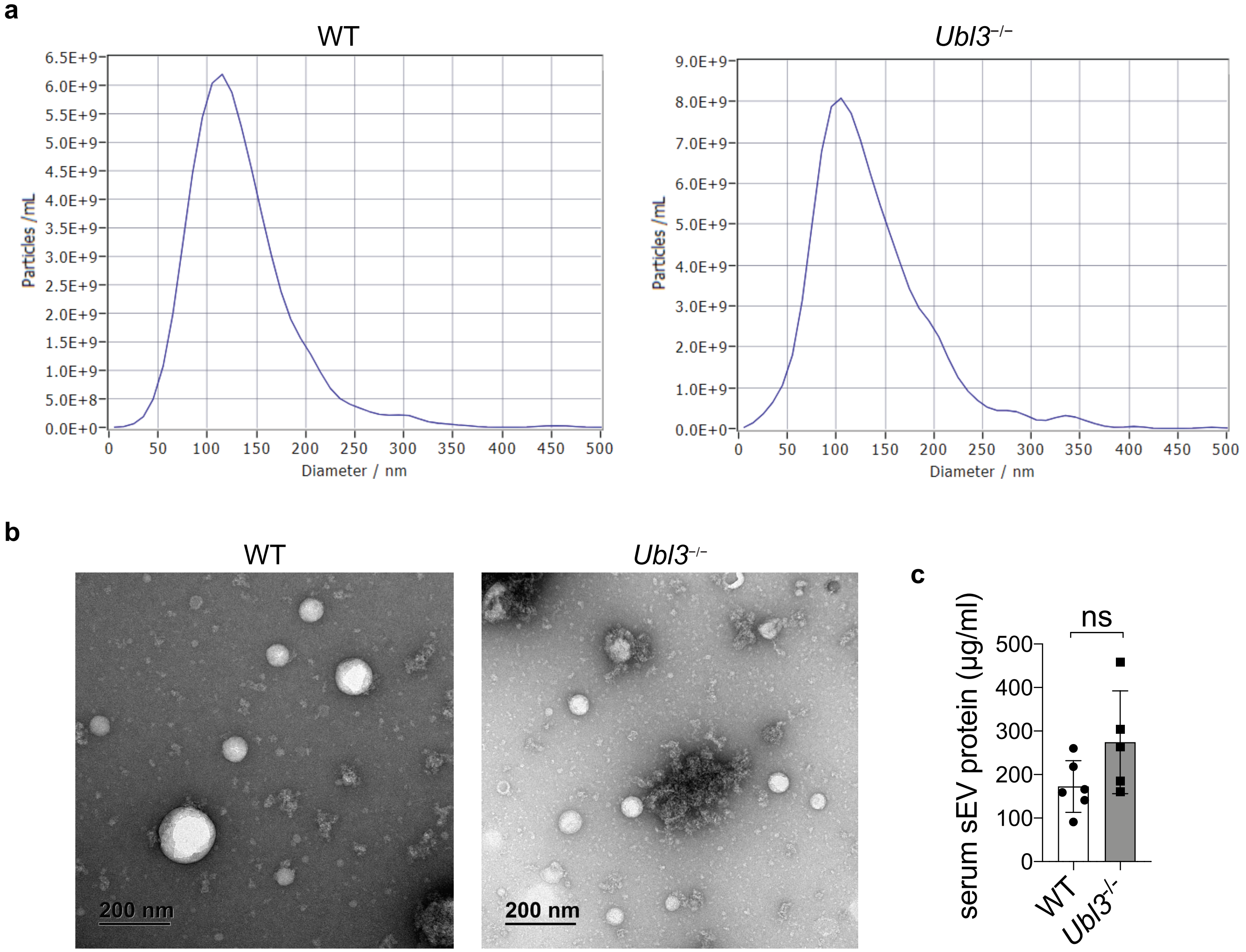

Supplementary figure 3. Loss of UBL3 does not affect protein content of serum small extracellular vesicles (sEVs). Small extracellular vesicles were isolated from serum of wild type or *Ubl3*$^{-/-}$ mice using differential ultracentrifugation. (a) Characterization of sEVs was performed by nanoparticle tracking analysis (NTA), where the size distributions indicate an enrichment of particles between 50-250 nm in both wild type sEV (left) and *Ubl3*$^{-/-}$ sEV (right). Average of 11 measurements is shown. (b) TEM analysis, where wild type sEV (left), and *Ubl3*$^{-/-}$ sEV (right) indicate the presence of vesicles possessing the size and characteristic cup shape of sEVs. Representative images are shown out of 14 (WT) or 25 (*Ubl3*$^{-/-}$) images taken in one experiment. (c) Protein content of isolated sEV was measured via BCA. Bars show mean ± SD with symbols representing individual mice, n = 6 for WT, 5 for *Ubl3*$^{-/-}$ examined across two independent experiments. Unpaired t test (two-sided), ns not significant..

Supplementary figure 4

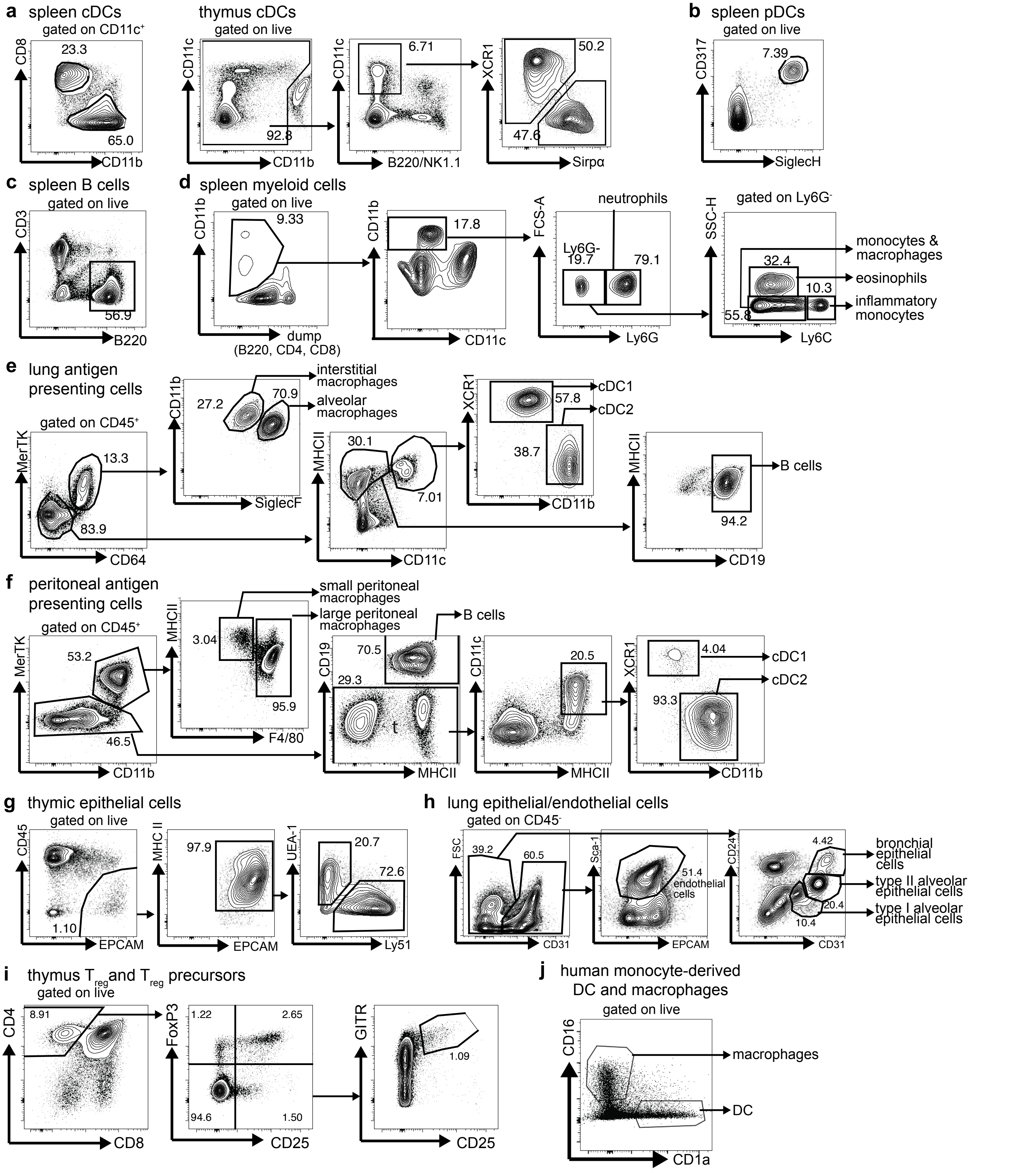

Supplementary figure 4. Gating strategies for flow cytometry analysis of primary immune cells. Isolated cell populations were gated on forward and side scatter, followed by exclusion of dead cells (propidium iodide positive). Example gating is shown for murine (a) spleen and thymus dendritic cells, (b) spleen plasmacytoid dendritic cells, (c) spleen B cells, (d) spleen myeloid cells, (e) lung antigen presenting cells, (f) peritoneal antigen presenting cells, (g) thymic epithelial cells, (h) lung epithelial cells, (i) thymus Treg and Treg precursors, and (j) human monocyte-derived macrophages and dendritic cells.

Supplementary figure 5

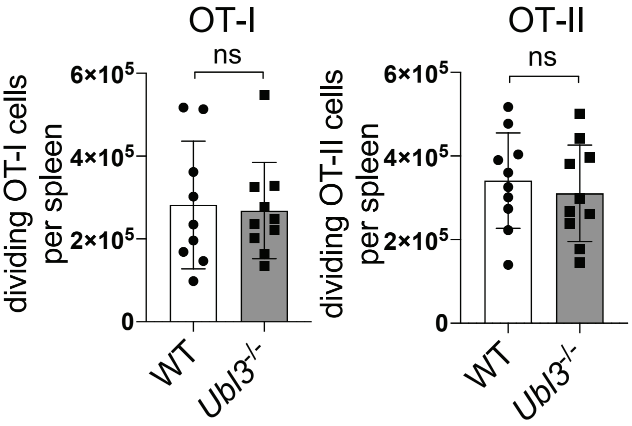

Supplementary figure 5. Lack of UBL3 does not affect OVA protein antigen presentation *in vivo*. Purified CellTrace Violet (CTV)-labelled OT-I and OT-II cells were adoptively transferred into *Ubl3*$^{-/-}$ and WT mice. 24 hours later, mice were injected with 50 µg ovalbumin (OVA), and spleens were harvested after 64 hours. Antigen presentation capacity was assessed by flow cytometric analysis of CTV-dilution to measure T cell proliferation. The number of dividing OT-I and OT-II was determined as described in Materials and Methods. Data from n = 9 for WT 10 for *Ubl3*$^{-/-}$ (OT-I) or n = 10 (OT-II) examined over two independent experiments, Symbols represent individual mice and bars show mean ± SD, ns not significant, unpaired t test (two-sided).

Supplementary figure 6

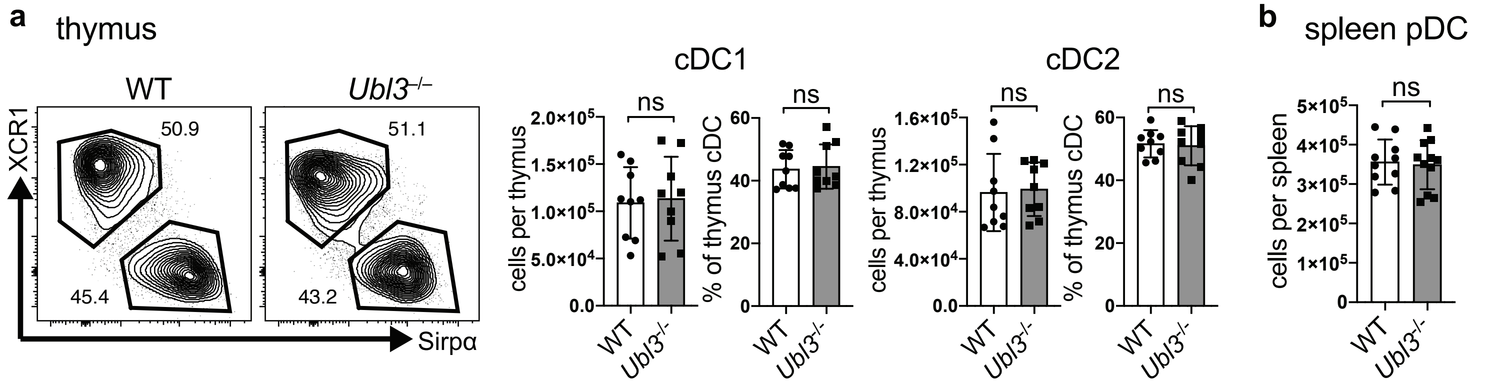

Supplementary figure 6. *Ubl3* deletion does not change thymic cDC or splenic pDC populations. Quantification of (a) thymus cDCs or (b) spleen pDCs of WT or *Ubl3*$^{-/-}$ mice, with cDC1 being identified as XCR1^{+}Sirpα^{-}, cDC2 identified as XCR1^{-}Sirpα^{+}, and pDCs identified as CD317^{+}SiglecH^{+}. For detailed gating strategy see Supplementary figure 2. Absolute cell numbers were determined as described in Materials and Methods. Data from (a) n = 9 mice examined over three independent experiments or (b) n = 10 for WT 11 for *Ubl3*$^{-/-}$ examined over two experiments, with symbols representing individual mice and bars mean ± SD, ns not significant, unpaired t test (two-sided).

Supplementary figure 7

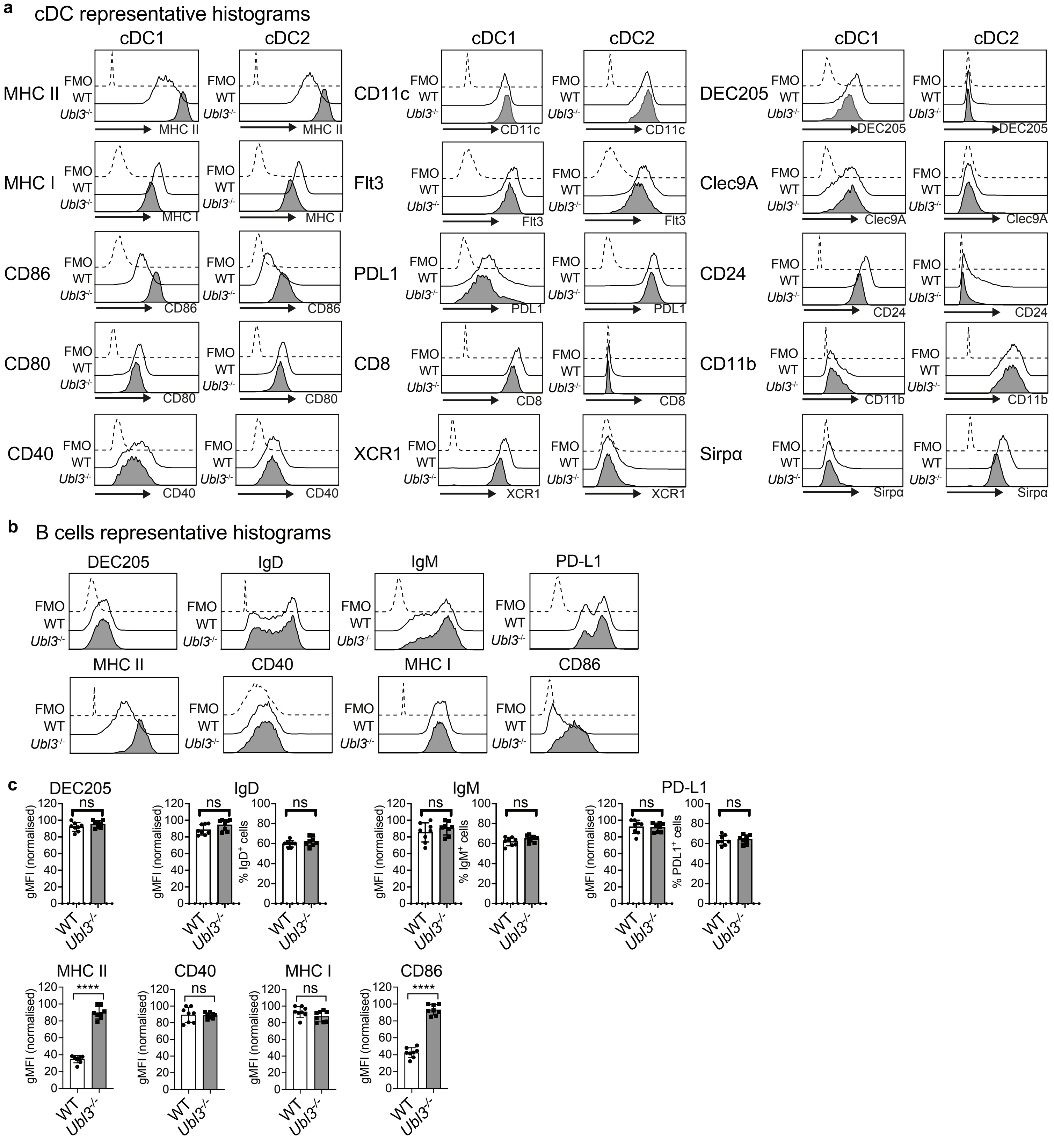

Supplementary figure 7. *Ubl3* affects cDC and B cell surface marker expression. Spleen (a) cDC1 and cDC2 or (b-c) B cells were enriched from WT and *Ubl3*$^{-/-}$ mice and stained for indicated surface markers. Shown are representative histograms (a-b) of marker staining for fluorescence minus one (FMO, dashed lines), wild type (WT, black lines) and *Ubl3*$^{-/-}$ samples (grey filled histograms), and (c) geometric mean fluorescence intensity for B220^{+}CD19^{+} B cells, with n = 8 mice examined over two independent experiments, with bars showing mean ± SD and symbols representing individual mice. **** P < 0.0001, ns not significant, unpaired t test (two-sided).

Supplementary figure 8

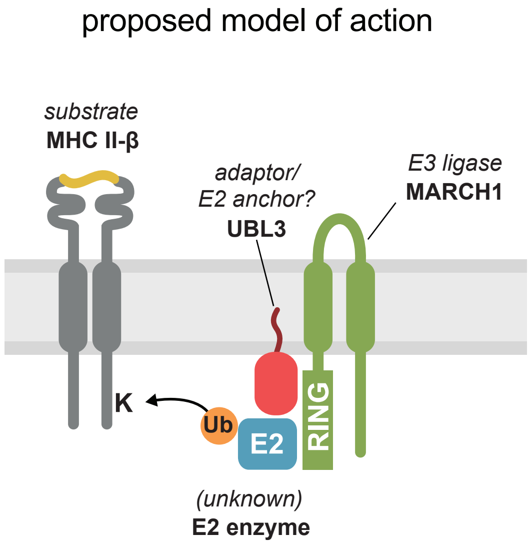

Supplementary figure 8. Proposed model of UBL3 regulation of MHCII. UBL3 (red) is anchored to in the cell membrane and interacts with MARCH1 (green). By binding an E2 enzyme (blue), it recruits it to the membrane and promotes transfer of ubiquitin (Ub) from the loaded E2 enzyme to the beta chain of MHC II (grey), resulting in ubiquitinated MHC II.

Supplementary Table 1. Enriched proteins in Myc-UBL3 expressing MutuDC compared to Δ*Ubl3*. Differential expression analysis was performed using the Bioconductor package limma, with adjustment for multiple testing using the Benjamini-Hochberg false discovery rate (FDR) method. Proteins were considered significantly enriched with an adjusted p-value cut-off <0.05 and log2 fold change >1 (see methods) over Δ*Ubl3* control. FC = fold change, asterisk denotes hits also detected in Myc-UBL3$_{C114S}$ samples.

FC	log_{10}(p value)	gene	name
**9.78**	3.78	**Ifi205a**	Interferon-activable protein 205-A
**9.58**	3.22	**Ubl3**	Ubiquitin-like protein 3
**9.19**	4.13	**Clasrp***	CLK4-associating serine/arginine rich protein
**9.06**	3.16	**Hdac1**	Histone deacetylase 1
**8.28**	4.44	**Utp18***	U3 small nucleolar RNA-associated protein 18 homologue
**7.89**	2.93	**Cct4**	T-complex protein 1 subunit delta
**7.78**	3.25	**Pdia6**	Protein disulfide-isomerase A6
**7.41**	3.05	**Lmnb1**	Lamin-B1
**7.11**	2.92	**Psap**	Prosaposin
**6.41**	3.03	**Vasp**	Vasodilator-stimulated phosphoprotein
**6.19**	3.78	**Sp110***	Sp110 nuclear body protein
**5.50**	3.15	**Tcp1**	T-complex protein 1 subunit alpha
**5.35**	2.95	**Nusap1**	Nucleolar and spindle-associated protein 1
**5.13**	3.41	**Hnrnpul2***	Heterogeneous nuclear ribonucleoprotein U-like protein 2
**4.72**	3.14	**Rab43**	Ras-related protein Rab-43
**4.38**	3.52	**Dpysl2**	Dihydropyrimidinase-related protein 2
**3.94**	3.27	**Gnl3l***	Guanine nucleotide-binding protein-like 3-like protein

Supplementary Table 2. Enriched proteins in Myc-UBL3$_{C114S}$ expressing MutuDC compared to Δ*Ubl3*. Differential expression analysis was performed using the Bioconductor package limma, with adjustment for multiple testing using the Benjamini-Hochberg false discovery rate (FDR) method. Proteins were considered significantly enriched with an adjusted p-value cut-off <0.05 and log2 fold change >1 (see methods) over Δ*Ubl3* control. FC = fold change

**FC**	**log$_{10}$(p value)**	**gene**	**name**
**3.48**	4.600	**Hnrnpul2**	Heterogeneous nuclear ribonucleoprotein U-like protein 2
**3.10**	4.29	**Sp110**	Sp110 nuclear body protein
**3.08**	4.01	**Clasrp**	CLK4-associating serine/arginine rich protein
**2.89**	4.26	**Utp18**	U3 small nucleolar RNA-associated protein 18 homologue
**2.08**	3.41	**Gnl3l**	Guanine nucleotide-binding protein-like 3-like protein

Supplementary Table 3. Flow cytometry antibodies used in this study.

Antigen/reagent	Reactivity	Supplier	Cat. No.	RRID	dilution
C3 (11H9) biotin	mouse	Novus	NB200-540B	AB_2744548	1:300
CD3 (KT3-1.1) FITC	mouse	WEHI antibody facility	N/A	N/A	1:200
CD3 (145-2C11) PE-Cy7	mouse	BioLegend	100320	AB_312685	1:200
CD4 (RM4-5) PE-Cy7	mouse	BioLegend	100528	AB_312729	1:400
CD8α (53-6.7) BV421	mouse	BioLegend	100738	AB_11204079	1:800
CD8α (53-6.7) APC	mouse	BioLegend	100712	AB_312751	1:400
CD11b (M1/70) PE	mouse	WEHI antibody facility	N/A	N/A	1:800
CD11b (M1/70) BV510	mouse	BioLegend	101245	AB_2561390	1:200
CD11b (M1/70) PE-Cy7	mouse	BioLegend	101216	AB_312799	1:200
CD11c (N418) PE-Cy7	mouse	BioLegend	117318	AB_493568	1:400
CD11c (N418) BV510	mouse	BioLegend	117338	AB_2562016	1:200
CD19 (1D3) FITC	mouse	WEHI antibody facility	N/A	N/A	1:800
CD19 (1D3) BB700	mouse	BD Biosciences	566411	AB_2744315	1:200
CD24 (M1/69) FITC	mouse	WEHI antibody facility	N/A	N/A	1:300
CD24 (M1/69) PE	mouse	WEHI antibody facility	N/A	N/A	1:100
CD25 (PC61) Biotin	mouse	WEHI antibody facility	N/A	N/A	1:200
CD31 (390) BUV805	mouse	BD Biosciences	741939	AB_2871251	1:200
CD40 (FGK45.5) APC	mouse	Miltenyi Biotec	130-102-547	AB_2660762	1:100
CD45 (30-F11) PerCP-Cy5.5	mouse	BioLegend	103132	AB_893340	1:100
CD45 (30-F11) BV786	mouse	BD Biosciences	564225	AB_2716861	1:200
CD45R/B220 (RA3-6B2) BV510	mouse	BioLegend	103248	AB_2650679	1:100
CD64 (X54-5/7.1) APC	mouse	BioLegend	139305	AB_11219205	1:200
CD80 (16-10A1) PE	mouse	BD Biosciences	553769	AB_395039	1:200
CD86 (GL1) APC	mouse	BioLegend	105012	AB_493342	1:400
CD86 (GL1) BV605	mouse	BioLegend	105037	AB_11204429	1:400
CD86 (GL1) FITC	mouse	BioLegend	105006	AB_313149	1:200
CD86 (GL1) BV650	mouse	BioLegend	105035	AB_11126147	1:200
CD135/Flt3 (A2F10) PE	mouse	BioLegend	135306	AB_1877217	1:400
CD172/Sirpα (P84) FITC	mouse	BioLegend	144005	AB_11204432	1:200
CD205/DEC205 (NLDC-145) PE	mouse	WEHI antibody facility	N/A	N/A	1:200
CD274/PD-L1 (10F.9G2) BV421	mouse	BioLegend	124315	AB_10897097	1:200
CD317/BST2 (927) FITC	mouse	BioLegend	127008	AB_2028462	1:200
CD326/EpCAM (G8.8) APC-Cy7	mouse	BioLegend	118218	AB_2098648	1:100
CD326/EpCAM (G8.8) PerCP-Cy5.5	mouse	BioLegend	118219	AB_2098647	1:100
CD357/GITR (DTA-1) APC	mouse	Thermo Fisher Scientific	17-5874-81	AB_469461	1:200

CD370/Clec9A (7H11) APC	mouse	BioLegend	143506	AB_2566380	1:200
F4/80 (F4/80) AF633	mouse	WEHI antibody facility	N/A	N/A	1:100
Foxp3 (FJK-16s) eF450	mouse	Thermo Fisher Scientific	48-5773-82	AB_1518812	1:800
IgD (11-26c.2a) FITC	mouse	BD Biosciences	553439	AB_394859	1:200
IgM (RMM-1) PE-Cy7	mouse	BioLegend	406515	AB_10690815	1:200
Ly6A.2/E.1/Sca-1 (D7) APC	mouse	BioLegend	108111	AB_313348	1:200
Ly6C.2 (5075-3.6) AF633	mouse	WEHI antibody facility	N/A	N/A	1:100
Ly6G (1A8) FITC	mouse	WEHI antibody facility	N/A	N/A	1:70
Ly6G (1A8) BUV395	mouse	BD Biosciences	563978	AB_2716852	1:200
Ly51 (6C3) FITC	mouse	BioLegend	108305	AB_313362	1:100
MerTK (2B10C42) BV421	mouse	BioLegend	151510	AB_2832533	1:200
MHC I H-2Kb (AF6-88.5) APC	mouse	BioLegend	116518	AB_10564404	1:400
MHC II I-A/I-E (M5/114) PE	mouse	WEHI antibody facility	N/A	N/A	1:300
MHC-II I-A/I-E (M5/114.15.2) AF700	mouse	BioLegend	107622	AB_493727	1:400
SiglecF (E50-2440) BV650	mouse	BD Biosciences	740557	AB_2740258	1:200
SiglecH (551) PE	mouse	BioLegend	129606	AB_2189147	1:200
XCR1 (ZET) PE	mouse	BioLegend	148203	AB_2563842	1:200
XCR1 (ZET) APC	mouse	BioLegend	148206	AB_2563932	1:400
Ulex Europaeus Agglutinin I (UEA-1), biotin	N/A	Vector Labs	B-1065-2	N/A	1:1200
CD1a (HI149) APC	human	BioLegend	300110	AB_314024	1:20
CD16 (3G8) FITC	human	BioLegend	302006	AB_314206	1:20
HLA-DR (LN3) APC-eFluor780	human	Thermo Fisher Scientific	47-9956-42	AB_1963603	1:20
CD86 (IT2.2) PE-Cy7	human	BioLegend	305422	AB_2074981	1:20
HLA-A,B,C (W6/32) PerCp-Cy5.5	human	BioLegend	311420	AB_10709152	1:20

Supplementary Table 4. Western blot antibodies used in this study.

Antibody	Supplier	Cat. No.	RRID	dilution
Rabbit anti-mouse Ubl3	Novus	ab113820	AB_10860670	1:1000
Mouse anti-human UBL3	LSbio	LS-C661402	AB_2885185	1:1000
Rabbit anti-Myc-tag (71D10)	Cell Signaling Technology	2278	AB_490778	1:1000
Rabbit anti-mouse actin (20-33)	Sigma-Aldrich	A5060	AB_476738	1:2000
Mouse anti-human actin (C4)	Millipore	MAB1501	AB_2223041	1:1000
Rat anti-ubiquitin (P4D1) HRP	Santa Cruz Biotechnology	sc-8017	AB_628423	1:1000
Rabbit anti-mouse MHCII beta chain (JV2)	WEHI antibody facility	N/A	N/A	1:1000

Supplementary Table 5. Immunofluorescence antibodies used in this study.

Antibody	Supplier	Cat. No.	RRID	dilution
anti-Myc-tag (clone 9E10)	BioLegend	626802	AB_2148451	1:200
anti-MHC II (clone M5/114)	WEHI antibody facility	N/A	N/A	1:200
goat anti-mouse Alexa Fluor 594	Thermo Fisher	A-11032	AB_2534091	1:1000
Alexa Fluor 647-conjugated avidin	produced in-house	N/A	N/A	1:800

Supplementary Methods

Isolation and protein content of small extracellular vesicles

Serum samples were diluted with the same volume of filtered (0.22 µm) PBS (no calcium, no magnesium) and centrifuged at 500g for 30 min at 4°C. Supernatant was collected, diluted with filtered PBS and centrifuged at 11,300 rpm for 45min, 4°C using a benchtop ultracentrifuge. Supernatants were collected and centrifuged at 100,000g for 90 minutes at 4°C. Supernatants were removed and pellets resuspended in PBS. For protein measurements, micro BCA™ Protein Assay Kit was utilised in 96-well format according to the user protocol. 150 µl of each sample was analysed in triplicate using the ClarioStar plate reader.

Nanoparticle tracking analysis of isolated EVs using Zetaview

Small EVs were analyzed for size and concentration via Zetaview installed with a 405 nm laser diode (Particle Metrix, PMX-120). Vesicles were prepared with a 1:2,000 (WT) and 1:4,000 (Ubl3) dilution in dPBS. For each cycle, 11 positions were taken with the following analysis parameters: camera sensitivity: 80, max area: 1000, min area: 5, min brightness: 30, min tracelength 15. dPBS was analyzed for background.

Transmission electron microscopy

For imaging Extracellular Vesicles (EV's) using transmission electron microscopy (TEM), EV's suspended in dPBS was applied to formvar-coated grid with heavy carbon coating after fixation in glutaraldehyde (2% v/w in H2O) for 30 mins room temperature. Samples were visualised by negative staining with uranyl acetate (2% w/v in H2O), with images captured using a Joel JEM-2100 electron microscope.

Mass spectrometry analysis

Δ*Ubl3* MutuDC or Δ*Ubl3* MutuDC expressing either Myc-UBL3$_{WT}$ or Myc-UBL3$_{C114S}$ were lysed in 1% Triton buffer on ice and nuclei removed by centrifugation at 14,000 × g at 4 °C. Postnuclear supernatants of 5 x 10^6 cells were incubated with anti-Myc-Tag (9B11) mouse mAb conjugated to magnetic beads (Cell Signaling Technologies) at 4 ºC, washed and eluted by acidification with 0.1% FA and denatured with 2,2,2-trifluoroethanol. Proteins were reduced with 12.5 mM tris(2-carboxyethyl)phosphine (TCEP), and digested with 0.25 mg proteomics grade Trypsin (Sigma-Aldrich) in 50 mM triethylammonium bicarbonate (TEAB)) overnight at 37 °C. Peptides were dried by SpeedVac, reconstituted in 2% acetonitrile and 0.05% trifluoroacetic acid, centrifuged for 10 minutes at 21,000 × g to remove particulate matter, and filtered over Vivacon 500 30kDa columns (Satrorius).

Samples were analyzed by liquid chromatography tandem mass spectrometry (LC-MS/MS) using Q-Exactive Plus mass spectrometer (Thermo Scientific) fitted with nanoflow reverse-phase high performance liquid chromatography (HPLC) (Ultimate 3000 RSLC, Dionex). The nano-LC system was equipped with an Acclaim Pepmap nano-trap column (Dionex – C18, 100 Å, 75 µm × 2 cm) and an Acclaim Pepmap RSLC analytical column (Dionex – C18, 100 Å, 75 µm × 50 cm). The peptide mix was loaded onto the enrichment (trap) column at an isocratic flow of 5 µL/min of 3% acetonitrile containing 0.1% formic acid. Eluents used were 0.1% v/v formic acid in water (solvent A) and 100% acetonitrile/0.1% formic acid v/v (solvent B). All spectra were acquired in positive mode with full scan MS spectra scanning from m/z 375-1400 at 70000 resolution with AGC target of 3e6 with a maximum accumulation time of 50 ms. The 15 most intense peptide ions with charge state ≥2-5 were isolated with an isolation window of 1.2 m/z and fragmented with a normalized collision energy of 30 at 17500 resolution with AGC target of 5e4 with a maximum accumulation time of 50ms. Underfill threshold was set to 2% for triggering of precursor for MS2. Dynamic exclusion was activated for 30s.

Raw MS files were processed with the MaxQuant software (ver. v1.6.17.0) using the integrated Andromeda search engine with false discovery ratio (FDR) < 0.01 at the protein and peptide levels against the mouse proteome (Uniprot proteome id UP000000589—Mus musculus, downloaded 09-03-2021). The search included variable modifications for oxidized methionine (M), and up to two missed cleavages were allowed for trypsin digestion. Protein quantification was performed with the included MaxLFQ option, and the resulting protein group output was analyzed using the LFQAnalyst portal[1] using the LFQ intensity values. Missing values were replaced by values drawn from a normal distribution of 1.8 standard deviations and a width of 0.3 for each sample (Perseus-type). Protein-wise linear models combined with empirical Bayes statistics were used for differential expression analysis using Bioconductor package Limma whereby the adjusted *p*-value cutoff was set at 0.05 and log2 fold change cutoff set at 1. The Benjamini-Hochberg (BH) method of FDR correction was used.

Supplementary References

1. Shah, A. D., Goode, R. J. A., Huang, C., Powell, D. R. & Schittenhelm, R. B. LFQ-Analyst: An Easy-To-Use Interactive Web Platform To Analyze and Visualize Label-Free Proteomics Data Preprocessed with MaxQuant. *J. Proteome Res.* **19**, 204–211 (2020).
