# Supplementary material for: Ubiquitin-like protein 3 (UBL3) is required for MARCH ubiquitination of major histocompatibility complex class II and CD86
Source: Nat Commun. 2022 Apr 11;13:1934. doi: 10.1038/s41467-022-29524-w (PMC9001657; doi:10.1038/s41467-022-29524-w)
Supplement: Supplementary file 2 — Reporting Summary [file 41467_2022_29524_MOESM2_ESM.pdf]

nature research

Corresponding author(s): Justine D. Mintern, Jose A. Villadangos

Last updated by author(s): Feb 8, 2022

Reporting Summary

Nature Research wishes to improve the reproducibility of the work that we publish. This form provides structure for consistency and transparency in reporting. For further information on Nature Research policies, see our Editorial Policies and the Editorial Policy Checklist.

Statistics

For all statistical analyses, confirm that the following items are present in the figure legend, table legend, main text, or Methods section.

n/a	Confirmed	
☐	☒	The exact sample size (n) for each experimental group/condition, given as a discrete number and unit of measurement
☐	☒	A statement on whether measurements were taken from distinct samples or whether the same sample was measured repeatedly
☐	☒	The statistical test(s) used AND whether they are one- or two-sided<br>*Only common tests should be described solely by name; describe more complex techniques in the Methods section.*
☒	☐	A description of all covariates tested
☐	☒	A description of any assumptions or corrections, such as tests of normality and adjustment for multiple comparisons
☐	☒	A full description of the statistical parameters including central tendency (e.g. means) or other basic estimates (e.g. regression coefficient) AND variation (e.g. standard deviation) or associated estimates of uncertainty (e.g. confidence intervals)
☐	☒	For null hypothesis testing, the test statistic (e.g. F, t, r) with confidence intervals, effect sizes, degrees of freedom and P value noted<br>*Give P values as exact values whenever suitable.*
☒	☐	For Bayesian analysis, information on the choice of priors and Markov chain Monte Carlo settings
☒	☐	For hierarchical and complex designs, identification of the appropriate level for tests and full reporting of outcomes
☒	☐	Estimates of effect sizes (e.g. Cohen's d, Pearson's r), indicating how they were calculated

Our web collection on statistics for biologists contains articles on many of the points above.

Software and code

Policy information about availability of computer code

Data collection	Flow cytometric data was obtained using FACSDiva version 9.0.1 (BD) and exported into FCS 3.0. Sequencing data collection for the CRISPR library were performed according to the instructions of the Illumina NextSeq 500 system. Western blot data was collected using ImageLab version 5.2.1 (Bio-Rad). Microscopy data was obtained using the LAS X software platform version 3.1.1. Mass Spectrometry data was obtained according to the instructions of the Q-Exactive system.
Data analysis	The CRISPR library screen was analysed in R using the edgeR package (https://bioconductor.org/packages/release/bioc/html/edgeR.html), flow cytometry data was analysed in Flowjo v9 and v10, microscopy data was analysed with the FIJI distrubition of ImageJ (2.0.0-rc-69/1.52p) (confocal microscopy), or Imaris v9.7 (proximity ligation assay). Statistical analysis of flow cytometric and western blot imaging data was performed in Prism versions v6, v8, and v9.0.0 (Graphpad). Mass Spectrometry data was analysed using MaxQuant software (ver. v1.6.17.0) and the LFQAnalyst portal (https://bioinformatics.erc.monash.edu/apps/LFQ-Analyst/ - accessed 14/07/2021)

For manuscripts utilizing custom algorithms or software that are central to the research but not yet described in published literature, software must be made available to editors and reviewers. We strongly encourage code deposition in a community repository (e.g. GitHub). See the Nature Research guidelines for submitting code & software for further information.

Data

Policy information about availability of data

All manuscripts must include a data availability statement. This statement should provide the following information, where applicable:

- Accession codes, unique identifiers, or web links for publicly available datasets
- A list of figures that have associated raw data
- A description of any restrictions on data availability

Source data underlying Figures 1a-b, 2b, 3a-b, 3d, 4a-i, 5a-b, 6a-d, 7a-e, 8, and Supplementary Figures 2a-c, 3c, 5, 6a-b, and 7c are provided as Source Data file. Proteomics data have been deposited to the ProteomeXchange Consortium via jPOST (accession codes JPST001444, PXD030790). The fasta file containing the mouse reference proteome used for the analysis of raw MS data using MaxQuant was retrieved from Uniprot (proteome id UP000000589, downloaded 09-03-2021).

Field-specific reporting

Please select the one below that is the best fit for your research. If you are not sure, read the appropriate sections before making your selection.

☒ Life sciences ☐ Behavioural & social sciences ☐ Ecological, evolutionary & environmental sciences

For a reference copy of the document with all sections, see nature.com/documents/nr-reporting-summary-flat.pdf

Life sciences study design

All studies must disclose on these points even when the disclosure is negative.

Sample size	All relevant sample sizes are described in the legends in the figure and/or the materials and methods section. Sample sizes were determined based on prior experiences with each of the experimental systems and their expected variability. No statistical testing was used to pre-determine saple size.
Data exclusions	After optimising experimental conditions, all data that met predetermined quality control expectations (e.g. sufficient staining, successful injection) were included.
Replication	Figure 1. The CRISPR KO library analysis in Fig 1 was done using 3 independent library transductions to ensure reproducibility. Additionally, this screen (using the GECKO v2 library) is a replication of a pilot experiment using an older generation library (unpublished, not included in the study) which showed similar results. All other experiments in the study were replicated by at least 2 independent experiments (except figures 2a, 3b, 4f,g,i, 6a(right),d, 7c and 8, and supplementary figures 2+3 as noted in the legends). All attempts at replication of experiments were successful. In addition, experiments using the UBL3cell lines Fig 3a+d were replicated successfully in a different clone of UBL3KO MutuDC cells.
Randomization	No randomisation was done as no experimental groups were used in this study.
Blinding	No blinding was used in our experiments, since the readouts were quantitative and not prone to subjective judgement of investirators. however the CRISPR KO screen was unbiased in nature.

Reporting for specific materials, systems and methods

We require information from authors about some types of materials, experimental systems and methods used in many studies. Here, indicate whether each material, system or method listed is relevant to your study. If you are not sure if a list item applies to your research, read the appropriate section before selecting a response.

Materials & experimental systems

n/a	Involved in the study
☐	☒ Antibodies
☐	☒ Eukaryotic cell lines
☒	☐ Palaeontology and archaeology
☐	☒ Animals and other organisms
☒	☐ Human research participants
☒	☐ Clinical data
☒	☐ Dual use research of concern

Methods

n/a	Involved in the study
☒	☐ ChIP-seq
☐	☒ Flow cytometry
☒	☐ MRI-based neuroimaging

Antibodies

Antibodies used	Following antibodies were used (with RRIDs provided for all commercially obtained antibodies).

Flow cytometry (mouse antigens):
C3 (11H9) biotin (Novus Cat# NB200-540B, RRID:AB_2744548) 1:300
CD3 (KT3-1.1) FITC (WEHI antibody facility, no Cat# available) 1:200
CD3 (145-2C11) PE-Cy7 (BioLegend Cat# 100320, RRID:AB_312685) 1:200
CD4 (RM4-5) PE-Cy7 (BioLegend Cat# 100528, RRID:AB_312729) 1:400
CD8α (53-6.7) BV421 (BioLegend Cat# 100738, RRID:AB_11204079) 1:800
CD8α (53-6.7) APC (BioLegend Cat# 100712, RRID:AB_312751) 1:400
CD11b (M1/70) PE (WEHI antibody facility) 1:800
CD11b (M1/70) BV510 (BioLegend Cat# 101245, RRID:AB_2561390) 1:200
CD11b (M1/70) PE-Cy7 (BioLegend Cat# 101216, RRID:AB_312799) 1:200
CD11c (N418) PE-Cy7 (BioLegend Cat# 117318, RRID:AB_493568) 1:400
CD11c (N418) BV510 (BioLegend Cat# 117338, RRID:AB_2562016) 1:200
CD19 (1D3) FITC (WEHI antibody facility) 1:800
CD19 (1D3) BB700 (BD Biosciences Cat# 566411, RRID:AB_2744315) 1:200
CD24 (M1/69) FITC (WEHI antibody facility) 1:300
CD24 (M1/69) PE (WEHI antibody facility) 1:100
CD25 (PC61) Biotin (WEHI antibody facility) 1:200
CD31 (390) BUV805 (BD Biosciences Cat# 741939, RRID:AB_2871251) 1:200
CD40 (FGK45.5) APC (Miltenyi Biotec Cat# 130-102-547, RRID:AB_2660762) 1:100
CD45 (30-F11) PerCP-Cy5.5 (BioLegend Cat# 103132, RRID:AB_893340) 1:100
CD45 (30-F11) BV786 (BD Biosciences Cat# 564225, RRID:AB_2716861) 1:200
CD45R/B220 (RA3-6B2) BV510 (BioLegend Cat# 103248, RRID:AB_2650679) 1:100
CD64 (X54-5/7.1) APC (BioLegend Cat# 139305, RRID:AB_11219205) 1:200
CD80 (16-10A1) PE (BD Biosciences Cat# 553769, RRID:AB_395039) 1:200
CD86 (GL1) APC (BioLegend Cat# 105012, RRID:AB_493342) 1:400
CD86 (GL1) BV605 (BioLegend Cat# 105037, RRID:AB_11204429) 1:400
CD86 (GL1) FITC (BioLegend Cat# 105006, RRID:AB_313149) 1:200
CD86 (GL1) BV650 (BioLegend Cat# 105035, RRID:AB_11126147) 1:200
CD135/Flt3 (A2F10) PE (BioLegend Cat# 135306, RRID:AB_1877217) 1:400
CD172/Sirpα (P84) FITC (BioLegend Cat# 144005, RRID:AB_11204432) 1:200
CD205/DEC205 (NLDC-145) PE (WEHI antibody facility) 1:200
CD274/PD-L1 (10F.9G2) BV421 (BioLegend Cat# 124315, RRID:AB_10897097) 1:200
CD317/BST2 (927) FITC (BioLegend Cat# 127008, RRID:AB_2028462) 1:200
CD326/EpCAM (G8.8) APC-Cy7 (BioLegend Cat# 118218, RRID:AB_2098648) 1:100
CD326/EpCAM (G8.8) PerCP-Cy5.5 (BioLegend Cat# 118219, RRID:AB_2098647) 1:100
CD357/GITR (DTA-1) APC (Thermo Fisher Scientific Cat# 17-5874-81, RRID:AB_469461) 1:200
CD370/Clec9A (7H11) APC (BioLegend Cat# 143506, RRID:AB_2566380) 1:200
F4/80 (F4/80) AF633 (WEHI antibody facility) 1:100
Foxp3 (FJK-16s) eF450 (Thermo Fisher Scientific Cat# 48-5773-82, RRID:AB_1518812) 1:800
IgD (11-26c.2a) FITC (BD Biosciences Cat# 553439, RRID:AB_394859) 1:200
IgM (RMM-1) PE-Cy7 (BioLegend Cat# 406515, RRID:AB_10690815) 1:200
Ly6A.2/E.1/Sca-1 (D7) APC (BioLegend Cat# 108111, RRID:AB_313348) 1:200
Ly6C.2 (5075-3.6) AF633 (WEHI antibody facility) 1:100
Ly6G (1A8) FITC (WEHI antibody facility) 1:70
Ly6G (1A8) BUV395 (BD Biosciences Cat# 563978, RRID:AB_2716852) 1:200
Ly51 (6C3) FITC (BioLegend Cat# 108305, RRID:AB_313362) 1:100
MerTK (2B10C42) BV421 (BioLegend Cat# 151510, RRID:AB_2832533) 1:200
MHC I H-2Kb (AF6-88.5) APC (BioLegend Cat# 116518, RRID:AB_10564404) 1:400
MHC II I-A/I-E (M5/114) PE (WEHI antibody facility) 1:300
MHC-II I-A/I-E (M5/114.15.2) AF700 (BioLegend Cat# 107622, RRID:AB_493727) 1:400
SiglecF (E50-2440) BV650 (BD Biosciences Cat# 740557, RRID:AB_2740258) 1:200
SiglecH (551) PE (BioLegend Cat# 129606, RRID:AB_2189147) 1:200
XCR1 (ZET) PE (BioLegend Cat# 148203, RRID:AB_2563842) 1:200
XCR1 (ZET) APC (BioLegend Cat# 148206, RRID:AB_2563932) 1:400

Validation

Flow cytometry: antibodies were titrated in house prior to using in experiments on C57BL/6 splenocytes (mouse antigens) or human PBMCs (human antigens). Additionally, all commercially obtained antibodies used were validated for flow cytometry by their respective manufacturers:

Mouse antigens
CD3 (145-2C11) PE-Cy7 (BioLegend Cat# 100320, RRID:AB_312685) https://www.biolegend.com/en-us/products/pe-cyanine7-anti-mouse-cd3epsilon-antibody-1899
CD4 (RM4-5) PE-Cy7 (BioLegend Cat# 100528, RRID:AB_312729) https://www.biolegend.com/en-us/products/pe-cyanine7-anti-mouse-cd4-antibody-1932
CD8α (53-6.7) BV421 (BioLegend Cat# 100738, RRID:AB_11204079) https://www.biolegend.com/en-us/products/brilliant-violet-421-anti-mouse-cd8a-antibody-7138
CD8α (53-6.7) APC (BioLegend Cat# 100712, RRID:AB_312751) https://www.biolegend.com/en-us/products/apc-anti-mouse-cd8a-antibody-150
CD11b (M1/70) BV510 (BioLegend Cat# 101245, RRID:AB_2561390) https://www.biolegend.com/en-us/products/brilliant-violet-510-anti-mouse-human-cd11b-antibody-7993
CD11b (M1/70) PE-Cy7 (BioLegend Cat# 101216, RRID:AB_312799) https://www.biolegend.com/en-us/products/pe-cyanine7-anti-mouse-human-cd11b-antibody-1921
CD11c (N418) PE-Cy7 (BioLegend Cat# 117318, RRID:AB_493568) https://www.biolegend.com/en-us/products/pe-cyanine7-anti-mouse-cd11c-antibody-3086
CD11c (N418) BV510 (BioLegend Cat# 117318, RRID:AB_2562016) https://www.biolegend.com/en-us/products/pe-cyanine7-anti-mouse-cd11c-antibody-3086

CD19 (1D3) BB700 (BD Biosciences Cat# 566411, RRID:AB_2744315) https://www.bdbiosciences.com/en-au/products/reagents/flow-cytometry-reagents/research-reagents/single-color-antibodies-ruo/bb700-rat-anti-mouse-cd19.566412
CD31 (390) BUV805 (BD Biosciences Cat# 741939, RRID:AB_2871251) https://www.bdbiosciences.com/en-au/products/reagents/flow-cytometry-reagents/research-reagents/single-color-antibodies-ruo/buv805-rat-anti-mouse-cd31.741939
CD40 (FGK45.5) APC (Miltenyi Biotec Cat# 130-102-547, RRID:AB_2660762) https://www.miltenyibiotec.com/AD-en/products/cd40-antibody-anti-mouse-fgk45-5.html#pure-functional-grade:1-mg-in-0-5-ml
CD45 (30-F11) PerCP-Cy5.5 (BioLegend Cat# 103132, RRID:AB_893340) https://www.biolegend.com/en-us/products/percp-cyanine5-5-anti-mouse-cd45-antibody-4264
CD45 (30-F11) BV786 (BD Biosciences Cat# 564225, RRID:AB_2716861) https://www.bdbiosciences.com/en-tw/products/reagents/flow-cytometry-reagents/research-reagents/single-color-antibodies-ruo/bv786-rat-anti-mouse-cd45.564225
CD45R/B220 (RA3-6B2) BV510 (BioLegend Cat# 103248, RRID:AB_2650679) https://www.biolegend.com/en-us/products/brilliant-violet-510-anti-mouse-human-cd45r-b220-antibody-7996
CD64 (X54-5/7.1) APC (BioLegend Cat# 139305, RRID:AB_11219205) https://www.biolegend.com/en-us/products/apc-anti-mouse-cd64-fcgammari-antibody-7874
CD80 (16-10A1) PE (BD Biosciences Cat# 553769, RRID:AB_395039) https://www.bdbiosciences.com/en-us/products/reagents/flow-cytometry-reagents/research-reagents/single-color-antibodies-ruo/pe-hamster-anti-mouse-cd80.553769
CD86 (GL1) APC (BioLegend Cat# 105012, RRID:AB_493342) https://www.biolegend.com/en-us/products/apc-anti-mouse-cd86-antibody-2896
CD86 (GL1) BV605 (BioLegend Cat# 105035, RRID:AB_11126147) https://www.biolegend.com/en-us/products/brilliant-violet-650-anti-mouse-cd86-antibody-7643
CD86 (GL1) FITC (BioLegend Cat# 105006, RRID:AB_313149) https://www.biolegend.com/en-us/products/fitc-anti-mouse-cd86-antibody-254
CD86 (GL1) BV650 (BioLegend Cat# 105035, RRID:AB_11126147) https://www.biolegend.com/en-us/products/brilliant-violet-650-anti-mouse-cd86-antibody-7643
CD135/Flt3 (A2F10) PE (BioLegend Cat# 135306, RRID:AB_1877217) https://www.biolegend.com/en-us/products/pe-anti-mouse-cd135-antibody-6173
CD172/Sirpα (P84) FITC (BioLegend Cat# 144005, RRID:AB_11204432) https://www.biolegend.com/en-us/products/fitc-anti-mouse-cd172a-sirpalpha-antibody-7829
CD274/PD-L1 (10F.9G2) BV421 (BioLegend Cat# 124315, RRID:AB_10897097) https://www.biolegend.com/en-us/products/brilliant-violet-421-anti-mouse-cd274-b7-h1-pd-l1-antibody-7250
CD317/BST2 (927) FITC (BioLegend Cat# 127008, RRID:AB_2028462) https://www.biolegend.com/en-us/products/fitc-anti-mouse-cd317-bst2-pdca-1-antibody-6349
CD326/EpCAM (G8.8) APC-Cy7 (BioLegend Cat# 118218, RRID:AB_2098648) https://www.biolegend.com/en-us/products/apc-cyanine7-anti-mouse-cd326-ep-cam-antibody-5577
CD326/EpCAM (G8.8) PerCP-Cy5.5 (BioLegend Cat# 118219, RRID:AB_2098647) https://www.biolegend.com/en-us/products/percp-cyanine5-5-anti-mouse-cd326-ep-cam-antibody-5602
CD357/GITR (DTA-1) APC (Thermo Fisher Scientific Cat# 17-5874-81, RRID:AB_469461) https://www.thermofisher.com/antibody/product/CD357-AITR-GITR-Antibody-clone-DTA-1-Monoclonal/17-5874-81
CD370/Clec9A (7H11) APC (BioLegend Cat# 143506, RRID:AB_2566380) https://www.biolegend.com/en-us/products/apc-anti-mouse-cd370-clec9a-dngr1-antibody-12367
Foxp3 (FJK-16s) eF450 (Thermo Fisher Scientific Cat# 48-5773-82, RRID:AB_1518812) https://www.thermofisher.com/antibody/product/FOXP3-Antibody-clone-FJK-16s-Monoclonal/48-5773-82
IgD (11-26c.2a) FITC (BD Biosciences Cat# 553439, RRID:AB_394859) https://www.bdbiosciences.com/en-us/products/reagents/flow-cytometry-reagents/research-reagents/single-color-antibodies-ruo/fitc-rat-anti-mouse-igd.553439
IgM (RMM-1) PE-Cy7 (BioLegend Cat# 406515, RRID:AB_10690815) https://www.biolegend.com/en-us/products/apc-cyanine7-anti-mouse-igm-6957
Ly6A.2/E.1/Sca-1 (D7) APC (BioLegend Cat# 108111, RRID:AB_313348) https://www.biolegend.com/en-us/products/apc-anti-mouse-ly-6a-e-sca-1-antibody-225
Ly6G (1A8) BUV395 (BD Biosciences Cat# 563978, RRID:AB_2716852) https://www.bdbiosciences.com/en-us/products/reagents/flow-cytometry-reagents/research-reagents/single-color-antibodies-ruo/buv395-rat-anti-mouse-ly-6g.563978
Ly51 (6C3) FITC (BioLegend Cat# 108305, RRID:AB_313362) https://www.biolegend.com/en-us/products/fitc-anti-mouse-ly-51-antibody-177
MerTK (2B10C42) BV421 (BioLegend Cat# 151510, RRID:AB_2832533) https://www.biolegend.com/en-us/products/brilliant-violet-421-anti-mouse-mertk-mer-antibody-18808
MHC I H-2Kb (AF6-88.5) APC (BioLegend Cat# 116518, RRID:AB_10564404) https://www.biolegend.com/en-us/products/apc-anti-mouse-h-2kb-antibody-6573
MHC-II I-A/I-E (M5/114.15.2) AF700 (BioLegend Cat# 107622, RRID:AB_493727) https://www.biolegend.com/en-us/products/alexa-fluor-700-anti-mouse-i-a-i-e-antibody-3413
SiglecF (E50-2440) BV650 (BD Biosciences Cat# 740557, RRID:AB_2740258) https://www.bdbiosciences.com/en-us/products/reagents/flow-cytometry-reagents/research-reagents/single-color-antibodies-ruo/bv650-rat-anti-mouse-siglec-f.740557
SiglecH (551) PE (BioLegend Cat# 129606, RRID:AB_2189147) https://www.biolegend.com/en-us/products/pe-anti-mouse-siglec-h-antibody-5178
XCR1 (ZET) PE (BioLegend Cat# 148203, RRID:AB_2563842) https://www.biolegend.com/en-us/products/pe-anti-mouse-rat-xcr1-antibody-10217
XCR1 (ZET) APC (BioLegend Cat# 148206, RRID:AB_2563932) https://www.biolegend.com/en-us/products/apc-anti-mouse-rat-xcr1-antibody-10222

Human antigens:
CD1a (HI149) APC (BioLegend Cat# 300110, RRID:AB_314024) https://www.biolegend.com/en-us/products/apc-anti-human-cd1a-antibody-703
CD16 (3G8) FITC (BioLegend Cat# 302006, RRID:AB_314206) https://www.biolegend.com/en-us/products/fitc-anti-human-cd16-antibody-567
HLA-DR (LN3) APC-eFluor780 (Thermo Fisher Scientific Cat# 47-9956-42, RRID:AB_1963603) https://www.thermofisher.com/antibody/product/HLA-DR-Antibody-clone-LN3-Monoclonal/47-9956-42
CD86 (IT2.2) PE-Cy7 (BioLegend Cat# 305422, RRID:AB_2074981) https://www.biolegend.com/en-us/products/pe-cyanine7-anti-human-cd86-antibody-3961

HLA-A,B,C (W6/32) PerCp-Cy5.5 (BioLegend Cat# 311420, RRID:AB_10709152) https://www.biolegend.com/en-us/products/percp-cyanine5-5-anti-human-hla-a-b-c-antibody-7057

Western blot: The rabbit antiserum against MHC II-beta (JV2, WEHI antibody facility) was generated against the C-terminal tail of MHC II-beta and validated by immunoblotting using WT and I-Aa-/- mouse splenocytes. The rabbit antibody against UBL3 (Abcam) was validated by Western Blot using WT and Ubl3-/- mouse spleen dendritic cells. Commercially sourced antibodies were also validated for Western Blot by their respective manufacturers:

Rabbit anti-mouse Ubl3 (Abcam Cat# ab113820, RRID:AB_10860670) http://www.abcam.com/ubl3-antibody-ab113820-references.html
Mouse anti-human UBL3 (LSbio Cat# LS-C661402, RRID:AB_2885185) https://www.lsbio.com/antibodies/ubl3-antibody-elisa-wb-western-ls-c661402/675370
Rabbit anti-Myc-tag (71D10) (Cell Signaling Technology Cat# 2278, RRID:AB_490778) https://www.cellsignal.com/products/primary-antibodies/myc-tag-71d10-rabbit-mab/2278
Rabbit anti-mouse actin (20-33) (Sigma-Aldrich Cat# A5060, RRID:AB_476738) https://www.sigmaaldrich.com/AU/en/product/sigma/a5060
Mouse anti-human actin (C4) (Millipore Cat# MAB1501, RRID:AB_2223041) https://www.merckmillipore.com/DE/de/product/Anti-Actin-Antibody-clone-C4,MM_NF-MAB1501
Rat anti-ubiquitin (P4D1) HRP (Santa Cruz Biotechnology Cat# sc-8017, RRID:AB_628423) https://www.scbt.com/p/ubiquitin-antibody-p4d1
Rabbit anti-mouse MHCII beta chain (JV2) (WEHI antibody facility)

Immunofluorescence antibodies were validated in house by microscopy using the DC1940 mouse cell line. Commercially obtained antibodies used were also validated for immunofluorescence by their respective manufacturers:
mouse mAb against Myc-tag (clone 9E10) (BioLegend Cat# 626802, RRID: AB_2148451) https://www.biolegend.com/en-us/products/purified-anti-c-myc-antibody-2873.

Eukaryotic cell lines

Policy information about cell lines

Cell line source(s)	MutuDC 1940 cells were a gift from Hans Acha-Orbea, HEK293 and 293FT cells for lentivirus production were sourced from ATCC.
Authentication	Cell lines were authenticated by flow cytometry for expression of expected markers, and morphological analysis.
Mycoplasma contamination	All cell lines were routinely tested for mycoplasma and were negative.
Commonly misidentified lines (See ICLAC register)	None were used.

Animals and other organisms

Policy information about studies involving animals; ARRIVE guidelines recommended for reporting animal research

Laboratory animals	C57BL/6J (H-2b), Marchf1−/−, Marchf8−/−, and Ubl3−/− age and sex matched mice (either female or male) were used at 6-12 weeks of age. Ubl3-/- mice were generated at the Melbourne Advanced Genome Editing Centre (MAGEC) facility via CRISPR/Cas9 system as described in Methods and Figure 2a. For other strains see methods for references.
Wild animals	No wild animals were used.
Field-collected samples	No field collected samples were used.
Ethics oversight	Experimental procedures were approved by the Animal Ethics Committee of the University of Melbourne (protocol no. 1714375).

Note that full information on the approval of the study protocol must also be provided in the manuscript.

Flow Cytometry

Plots

Confirm that:

☒ The axis labels state the marker and fluorochrome used (e.g. CD4-FITC).

☒ The axis scales are clearly visible. Include numbers along axes only for bottom left plot of group (a 'group' is an analysis of identical markers).

☒ All plots are contour plots with outliers or pseudocolor plots.

☒ A numerical value for number of cells or percentage (with statistics) is provided.

Methodology

Sample preparation	For isolation of DC or trogocytic B cells from mouse spleen or thymus, organs were finely chopped and digested by manual agitation in the presence of DNase I and collagenase type 3 (please see methods section for details and references), and cell clusters were disrupted by addition of 10 mM EDTA. Light density cells were isolated by density gradient separation in 1.077 g/cm3 Nycodenz, collected cells were washed and either analysed by flow cytometry (thymus DC, trogocytic B cells) or further enriched (spleen) by incubation in a depletion cocktail, washing with EDTA-BSS+2%FCS, and incubated with BioMag anti-rat IgG-coupled magnetic beads (Qiagen). The DC-enriched supernatant was recovered by magnetic separation.<br><br>Mouse spleen, blood and peritoneal cavity cells were treated with red cell removal buffer, and washed with EDTA-BSS+2% FCS prior to staining.<br><br>Mouse thymic epithelial cells were enriched as previously described (please see methods section for details and references). In short, single thymi were clipped and mechanically dispersed, followed by sequential digestion with DNase I and liberase. filtered over a nylon mesh and washed with EDTA-BSS+2%FCS prior to staining.<br><br>For analysis of mouse lung cells, right ventricles were perfused with PBS and the lungs were collected, finely chopped and digested in liberase and DNAse I at 37ºC. Tissue was mechanically disrupted by pipetting, filtered over a 70µm strainer, treated with red cell removal buffer, and washed with EDTA-BSS+2%FCS prior to staining.<br><br>For thymic Treg analysis, thymi were mashed through a 40µm strainer, washed with EDTA-BSS+2%FCS, and stained with antibodies against surface markers, fixed and permeabilised, before staining for Foxp3.<br><br>For human monocyte analysis, buffy coats from healthy donors were obtained from Etablissement Français du Sang (Paris) in accordance with INSERM ethical guidelines. Peripheral Blood Mononuclear Cells (PBMC) were prepared by centrifugation on a Ficoll gradient. Blood CD14+ monocytes were isolated from healthy donors' PBMC by positive selection using magnetic beads.
Instrument	human samples: FACSVerse (BD), mouse lung samples: CytoFLEX LX (Beckman Coulter), all other samples: LSR Fortessa (BD)
Software	Data was acquired using FACS Diva (BD) and analysed using Flowjo (Treestar)
Cell population abundance	Mouse cDC used for MHCII immunoprecipitation were 70-85% CD11c+ pure as assessed by flow cytometry. Human monocytes used for Western blotting were 95-98% CD14+CD16- as assessed by flow cytometry.
Gating strategy	1. SSC-A vs FSC-A to exclude cell debris<br>2. FSC-H vs FSC-A to include single cells<br>3. Viability dye (Either propidium iodide, DAPI or fixable viability dye eFluor780) vs Fsc-A to exclude dead cells<br>4. Further gating is described in Supplementary Figure 4

☒ Tick this box to confirm that a figure exemplifying the gating strategy is provided in the Supplementary Information.
